# HaploCoV: unsupervised classification and rapid detection of novel emerging variants of SARS-CoV-2

**DOI:** 10.1038/s42003-023-04784-4

**Published:** 2023-04-22

**Authors:** Matteo Chiara, David S. Horner, Erika Ferrandi, Carmela Gissi, Graziano Pesole

**Affiliations:** 1 Department of Biosciences, University of Milan, Milan, Italy; 2 Institute of Biomembranes, Bioenergetics and Molecular Biotechnologies, Consiglio Nazionale delle Ricerche, Bari, Italy; 3 Department of Biosciences, Biotechnology and Environment, University of Bari “A. Moro”, Bari, Italy

**Keywords:** Comparative genomics, Computational biology and bioinformatics, Pathogens

## Abstract

Accurate and timely monitoring of the evolution of SARS-CoV-2 is crucial for identifying and tracking potentially more transmissible/virulent viral variants, and implement mitigation strategies to limit their spread. Here we introduce HaploCoV, a novel software framework that enables the exploration of SARS-CoV-2 genomic diversity through space and time, to identify novel emerging viral variants and prioritize variants of potential epidemiological interest in a rapid and unsupervised manner. HaploCoV can integrate with any classification/nomenclature and incorporates an effective scoring system for the prioritization of SARS-CoV-2 variants. By performing retrospective analyses of more than 11.5 M genome sequences we show that HaploCoV demonstrates high levels of accuracy and reproducibility and identifies the large majority of epidemiologically relevant viral variants - as flagged by international health authorities – automatically and with rapid turn-around times.

Our results highlight the importance of the application of strategies based on the systematic analysis and integration of regional data for rapid identification of novel, emerging variants of SARS-CoV-2. We believe that the approach outlined in this study will contribute to relevant advances to current and future genomic surveillance methods.

## Abstract (web summary)

HaploCoV is a software framework that enables users to explore the genetic diversity of SARS-CoV-2 variants in time and across different geographic locales, as well as prioritize emerging variants for further investigation.

## Introduction

The timely and accurate detection of emerging variants of a pathogen is one of the key goals of genomic surveillance and represents the first line of defense for limiting the spread of more severe/contagious forms of an infectious disease^1^. During the COVID-19 pandemic, genome sequencing has allowed the identification of SARS-CoV-2 lineages associated with distinct epidemiological events and geographic regions. Throughout the COVID-19 pandemic millions of SARS-CoV-2 genomic sequences from 206 distinct countries have been made available through the GISAID EpiCoV database^2^.

Pango, a sophisticated phylogenetic classification system developed by Rambaut et al.^3^, has been endorsed by international health authorities and represents the gold standard for the classification of SARS-CoV-2 viral lineages^4^. This system implements a hierarchical four-level nomenclature. Distinct lineages are established based on phylogenetic evidence and important epidemiological and biological characteristics. As of 10/06/2022 Pango delineated 1720 distinct SARS-CoV-2 lineages. Among these, 424 lineages have been under scrutiny by international health authorities due to their widespread and sustained circulation, coupled with the presence of genomic variants associated with increased infectivity and/or reduced neutralization by antibodies in the Spike glycoprotein^5–7^. Collectively these lineages are known under the acronyms VOC (Variant of Concern), VOI (Variant of Interest) and VUM (Variant under Monitoring)^8^ and represent a source of concern for the effectiveness of measures for the containment of COVID-19^9^. SARS-CoV-2 VOCs, VOIs and VUMs show highly biased geographic distributions, and, until recently, were typically designated by the country of first isolation (Supplementary Data 1). While most lineages in the Pango nomenclature are compact in size (median 179 distinct genomes), the 10 most numerous designations incorporate 52.45% of the available genomes (our statistics), suggesting that a limited number of highly successful viral variants account for the large majority of the cases of COVID-19 worldwide.

Several lines of evidence suggest that sampling of SARS-CoV-2 genomic sequences throughout the COVID-19 pandemic might present substantial geographic biases^10^ due to various factors including: local incidence of COVID-19^11^, access to state of the art molecular diagnostic tests and sequencing facilities^12,13^, bottlenecks in bioinformatics analyses and sharing of data^14,15^, as well as the application of different rationales and criteria by local health authorities in the implementation of genomic surveillance. These biases pose relevant questions about the accuracy and completeness of estimates of the prevalence of SARS-CoV-2 lineages, with possible implications for both genomic surveillance and the rapid identification of relevant novel variants.

We and others^16,17^ have recently proposed strategies based on the phenetic clustering of highly prevalent genomic variants (with frequency, AF, >1%) for capturing emerging viral genetic diversity in SARS-CoV-2. Here we extend our approach to develop a general and fully automated framework for the rapid delineation and prioritization of SARS-CoV-2 variants.

The vast number of SARS-CoV-2 genomic sequences collected during the COVID-19 pandemic and the observed patterns of genomic diversity and viral evolution pose a considerable challenge for the classification and tracking of SARS-CoV-2 variants. While in principle genomic data allow a capillary mapping of the spread of each distinct isolate across and between geographic locales, the large majority of viral strains typically differ by a limited number of genomic variants and are unlikely to demonstrate epidemiologically/pathologically distinct characteristics. In the light of these considerations, we propose an intermediate approach wherein phenetic clustering of viral genomes is applied to stratify viral isolates according to carefully selected criteria; and subsequently genomic features are used to infer/prioritize novel variants of potential epidemiological relevance by leveraging knowledge of the characteristics of current and past VOC/VOI/VUM. Moreover, genomic surveillance of SARS-CoV-2 (or any pathogen) is a dynamic process and epidemiologically important viral lineages/characteristics might change over time. Accordingly, to facilitate expert manual intepretation of results, automated systems for the analysis and classification of pathogen genomic data should, in our opinion, feature simple and human interpretable rules.

To illustrate the applicability and potential advantages of this approach, we developed HaploCoV, a standalone software package for the identification and prioritization of SARS-CoV-2 viral variants that can integrate/complement any classification/nomenclature system. By applying our software to the complete collection of 11.5 M SARS-CoV-2 genome sequences, as available on 10/06/2022, we demonstrate that our method can identify viral variants flagged by international health authorities in an unsupervised manner, with very short turn-around times and high accuracy. In addition, we highlight several genetic variants with highly biased geographical distribution, which are not captured by current nomenclature standards. Finally, we present an effective computational strategy for the rapid and automatic prioritization of variants of SARS-CoV-2 with potentially relevant epidemiological features and provide specific observations concerning the recurrent mutations associated with these viral variants.

Taken together, we believe that the main results and the approach outlined in this work can provide a substantial contribution to current and future methods for genomic surveillance strategies of human pathogens. A collection of tools and software for the automated analysis of SARS-CoV-2 genomic sequence data is made publicly available at https://github.com/matteo14c/HaploCoV.

## Results

### Biased sampling and geographic diversity in SARS-CoV-2 genomic data

According to our analyses, submissions of publicly available SARS-CoV-2 genomic sequences from the USA and the UK accounted for more than 55.2% of the genomic sequences available in the GISAID EpiCoV database. As outlined in Supplementary Fig. 1a, the total number of genomic sequences deposited from different macro-geographic areas was extremely variable, even through time. For many countries, sequence production did not correlate with the incidence of COVID-19 (Supplementary Fig. 1b) and the large majority of SARS-CoV-2 lineages identified by the Pango nomenclature were preferentially associated with macro-geographic areas that provided a higher contribution in terms of deposition of genomic sequences (Supplementary Fig. 2a). For example, while more than 1000 distinct lineages were detected in North America, central Europe and UK (NAnorth, Euc and EuUK in Supplementary Fig. 2a), the equivalent figure was below 400 for all the African macro-geographic regions (AfrCent, AfrEast, AfrN, AfrSouth, AfrW), indicating a potential sampling bias. Accordingly, a limited correlation was observed between the incidence of COVID-19 and the number of Pango lineages identified in different countries (Supplementary Fig. 2b).

**Fig. 1:**
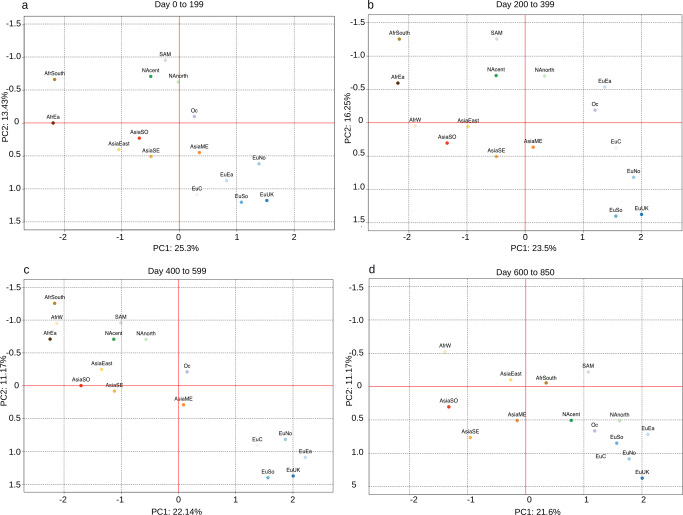
Principal component analysis of genomic variants frequencies in different geographic macro-areas. Four distinct time intervals are considered, with time T0 set at Time 0 = December 26th 2019: the day of reported isolation of the first SARS-CoV-2 genomic sequence. **a** from day 0 to 199. **b** from day 200 to 399. **c** from day 400 to 599, **d** from day 600 to 850. The following acronym are used for different geographic areas AfrCent Central Africa, AfrEast Eastern Africa, AfrN Northern Africa, AfrSouth Southern Africa, AfrW Western Africa, AsiaCent Central Asia, AsiaEast Eastern Asia, AsiaME Middle East, AsiaSE South Eastern Asia, AsiaSO Southern Asia, Euc Central Europe, EuEa Eastern Europe, EuNo Northern Europe, EuSo Southern Europe, EuUK United Kingdom, NAcent central America, NAnorth North America, Oc Oceania, SAM South America. See Supplementary Data 3 for the correspondence between geographic areas and countries.

A total of 238,287 distinct genomic variants (i.e., SNPs, rearrangements, and indels with respect to the reference genome) were recovered in the collection of SARS-CoV-2 genomic sequences analyzed in this work (Supplementary Data 2). Analyses of allele frequency (AF) distributions were performed on aggregated data, on overlapping intervals of 10 days starting from December 26th 2019, the reported day of isolation of the first sequenced SARS-CoV-2 genome. Consistent with previous reports^18,19^, only a limited number of genomic variants exhibited a prevalence of 1% or above in the global viral population at more than one time interval (745 = 0.31%, see HF_global in Supplementary Data 2), while the large majority (237,542 = 99.69%) never reached this minimum frequency threshold. Consistent with the observed biases in geographic sampling, when genomic sequences were partitioned according to macro-geographic regions (as defined in Supplementary Data 3) a more than 16 fold increase in the number of high frequency genomic variants was observed. Strikingly, a more than 40 fold increase was observed when country-level data were considered (HF genomic variants at global level: 785; at macro-geographic areas level: 13,413; at country level: 31,040; Supplementary Data 4). These observations suggest the circulation of a large number of viral lineages which are not prevalent globally, but might be specific to a country and/or macro-geographic area. Dimensionality reduction analyses on the complete collection of 238,287 genomic variants was performed by considering four distinct time intervals (Fig. 1a–d). Consistent with our previous observations, a clear separation between distinct macro-geographic areas was observed irrespective of the time interval considered, with the European and African regions showing the highest degree of separation on both axes. A clear geographic clustering was also observed when equivalent analyses were performed on single countries from which ≥5000 genomic sequences were available (Supplementary Fig. 3a–d). Interestingly, a neat separation between European and non-European macro-areas (and countries) was observed in the time interval from day 400 to 599 (Fig. 1c and Supplementary Fig. 3c), in the interval of time that corresponds with the rapid fixation of the Alpha VOC in many European countries^18^. Moreover, a generalized decrease in genomic diversity, indicated by a greater proximity of all points, is observed from day 600 to 850, the interval of time associated with the emergence and spread of the Delta^19^ and Omicron^20,21^ variants worldwide (Fig. 1c and Supplementary Fig. 3d).

Notably, 23,283 distinct genomic variants, corresponding to 9.77% of the total number, were found to display a significant bias in their geographic distribution (Benjamini corrected Fisher test *p* value ≤ 0.01). In addition, when genomic assemblies submitted from different countries were compared, we noticed that genome assembly size distributions (Supplementary Fig. 4) differed between submitting countries, resulting in a possible additional source of technical bias. For example, when the criteria established in Chiara et al.^16^ for the definition of high quality assemblies were applied, large discrepancies—from 99.17% (Greece) to 24.43% (Romania)—were observed in proportions of high quality genomic sequences produced by different countries (Supplementary Data 5).

Based on these observations we speculated that the observed discrepancies in the completeness and quality of genomic sequences, coupled with the highly uneven sampling of viral isolates at geographic level could pose a hindrance for the accurate and rapid identification of SARS-CoV-2 variants. Further analyses were undertaken to explore this hypothesis.

### A novel framework for the exploration of genomic diversity in SARS-CoV-2

Recently, we introduced a standalone system for the classification of genomic diversity in SARS-CoV-2^16^. Our method combined hierarchical clustering of phenetic patterns of presence/absence of high frequency genomic variants and a set of empirical rules to define distinct groups of viral haplotypes or haplogroups (HGs)^16^. HGs were established as unique, linear combinations of high frequency viral genomic variants. To be included in the system each HGs was required to incorporate at least 100 genomic sequences and be defined by a unique combination of 2 or more high frequency genomic variants. These rules were established by empirical analyses of SARS-CoV-2 evolutionary dynamics and based on the limited levels of diversity observed between closely related sequences. Although our method demonstrated to be highly reliable and specific^16^, its limited flexibility due to the application of arbitrarily established hard-thresholds—both for the inclusion/exclusion of genome sequences and the computation of allele frequency patterns—and limited possibility for integration with other widely adopted nomenclature systems represented important limitations. Moreover, since frequencies of genomic variants were estimated on the global viral population as a whole, systematic differences in the number of genomic sequences from distinct geographic areas, together with the observed discrepancies in their overall quality and completeness (see previous paragraph), could represent relevant issues for the prompt and accurate mapping and classification of SARS-CoV-2 genomic diversity.

In the light of these considerations we revised and extended our system for the analysis of genomic diversity in SARS-CoV-2 to: allow the exploration of patterns of genomic variation at different levels of geographic granularity (regions, countries and/or macro-geographic areas); facilitate the integration with nomenclature standards for the discovery of novel variants; and enable the definition of custom rules/criteria for the definition of high frequency genomic variants and the formation of designations/clades.

Given the potential of low quality genomes for affecting estimates of genomic variants frequencies and the observation that the large majority (87%) of low quality sequences were incomplete either at 5′ and/or 3′ UTR, default criteria for inclusion of sequences in our analyses were relaxed (see Materials and Methods). As a consequence, a more uniform proportion of high quality genomes was observed across different countries (see columns 2 and 3 in Supplementary Data 5).

To mitigate potential geographic biases, a novel standalone utility was developed to allow the computation of frequencies for distinct macro-geographic regions (as defined in Supplementary Data 3) and countries, moreover hard-thresholds for the definition of high frequency genomic variants and for their persistence in the viral population were removed, and users are now allowed to specify the values that better suit their specific use case. Finally, our approach was extended to allow its integration within any other classification system of choice. Metadata, including the place and date of isolation and the group/lineages associated with each sequence, must be provided in the form of a tabular file. Phenetic profiles of presence/absence of genomic variants are derived automatically; subsequently, high frequency genomic variants are established and the classification system is augmented, according to the user established criteria.

**Fig. 2:**
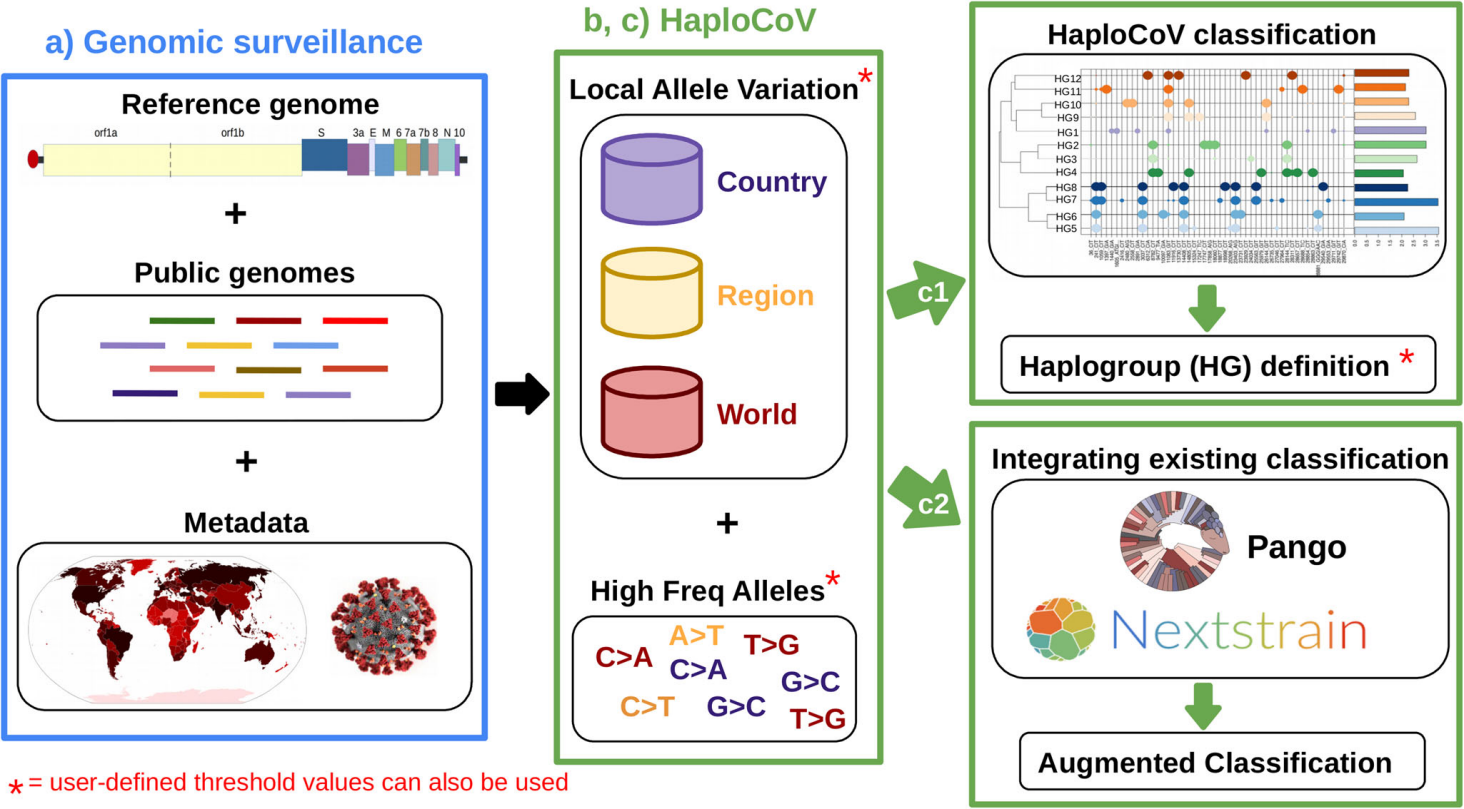
Workflow and potential applications of HaploCoV. **a** SARS-CoV-2 genomic surveillance: genome sequences and associated metadata, obtained from publicly available repositories and/or other resources, are consolidated into a local database. **b** HaploCoV workflow: Firstly, genomic sequences are compared with the reference genome assembly of SARS-CoV-2 to derive a complete collection of genomic variants. Subsequently allele frequencies are computed and a collection of high frequency genomic variants is obtained. Finally, phenetic clustering of high frequency genomic variants is applied: to (**c1**) derive HGs of SARS-CoV-2 based on a user defined minimum phenetic distance (i.e., groups that differ by more than a user defined number of genomic variants); and/or (**c2**) to complement an existing classification system by applying phenetic clustering to pre-defined groups/lineages. The illustration of the SARS-CoV-2 structure included in the figure was obtained from the Public Health Image Library (PHIL) by the Centers for Disease Control and Prevention (CDC), at https://phil.cdc.gov/Details.aspx?pid=23312.

HaploCoV, our novel software suite for the analysis of SARS-CoV-2 genomic sequences, enables the rapid execution of custom analyses for discovery of patterns of regional variation in SARS-CoV-2. Results can be seamlessly integrated within any pre-existing nomenclature/classification to facilitate the discovery, functional annotation and prioritization of novel emerging variants of the virus. An overview of the workflow and of the potential applications is depicted in Fig. 2.

#### HaploCoV can identify all current VOC, VOI and VUM variants in an unsupervised manner

To demonstrate its application and the reproducibility of the results, HaploCoV was applied to the complete collection of 11.5 M SARS-CoV-2 genomic sequences as available on June 10th 2022. A total of 24 distinct combinations of threshold values (4 minimum allele frequency cut-offs and 6 distinct cut-offs for the persistence in the viral population) were compared to derive the most appropriate settings for the delineation of HGs by HaploCoV Distinct combinations of values were evaluated both with respect to the ability to minimize the total number of HGs formed and to the ability to reconstruct groups corresponding to the current and past VOCs/VOIs/VUMs in a timely and accurate manner. Results are summarized in Table 1.

**Table 1:** Combinations of parameters used in the optimization of phenetic clustering of SARS-CoV-2 genomes.

Freq. Threshold	Pers. Threshold	VOC > 1HG/Tot VOC	VOC > 1HG/ Tot VOI	VUM > 1HG/Tot VUM	Tot HGs
0.5	10	5/5	9/9	14/14	7936
0.5	15	5/5	9/9	14/14	5854
0.5	25	5/5	9/9	14/14	4799
0.5	50	4/5	8/9	10/14	4076
0.5	75	3/5	7/9	9/14	3960
0.5	100	2/5	4/9	8/14	2957
1	10	5/5	9/9	14/14	2979
1	15	5/5	9/9	14/14	1571
1	25	5/5	9/9	13/14	976
1	50	5/5	8/9	13/14	863
1	75	4/5	7/9	11/14	801
1	100	2/5	5/9	8/14	736
2.5	10	5/5	8/9	11/14	1360
2.5	15	5/5	7/9	10/14	1182
2.5	25	5/5	7/9	10/14	938
2.5	50	2/5	6/9	10/14	801
2.5	75	2/5	5/9	4/14	736
2.5	100	2/5	4/9	4/14	583
5	10	5/5	7/9	6/14	768
5	15	2/5	4/9	6/14	659
5	25	2/5	3/9	4/14	482
5	50	1/5	2/9	4/14	424
5	75	1/5	1/9	2/14	401
5	100	1/5	1/9	2/14	388

Freq. Threshold: minimum frequency threshold. Pers. Threshold: minimum number of days above the minimum AF threshold. VOC > 1HG/Tot VOC: number of VOC for which at least 1 HG was designated compared to the total number of VOC. VOC > 1HG/Tot VOI: number of VOI for which at least 1 HG was designated compared to the total number of VOI. VUM > 1HG/Tot VUM: number of VUM for which at least 1 HG was designated compared to the total number of VUM. Tot HGs Total number of HGs identified.

Based on our criteria, a minimum AF of 1% and a persistence of 15 or more days were selected as the most appropriate settings for the definition of high frequency genomic variants in HaploCoV. A total of 1571 distinct HGs were formed under these settings, compared with the 1290 HGs defined by applying the original settings of Chiara et al. (2021). Importantly, while the number of distinct designations was lower than the number of lineages in the Pango nomenclature (1571 versus 1720), each of the 28 VOC/VOI/VUM was associated with at least one distinct designation. Moreover, as reported in Table 2, for all of the 20 VOC/VOI/VUM with a known geographic origin, an equivalent HG was designated by HaploCoV within a time interval compatible with their recognition by international health authorities, suggesting that our approach could identify potential VOC/VOI/VUM in a timely and accurate manner. Indeed, the average time from HG detection to VOC/VOI/VUM recognition was 70 days (last columns in Table 3).

**Table 2:** Time from emergence of a VOI/VOC/VUM to formation of an HG in HaploCoV.

Pango lineage	VOC/VOI/VUM	Time first detected	Days from detection to HG	Days from detection to VOC/VOI/VUM	Days from HG designation to VOC/VOI/VUM
B.1.1.7	VOC	Sept 2020	27	75	48
P.1	VOC	Nov 2020	25	36	11
B.1.351	VOC	May 2020	161	198	37
B.1.617.1	VOI	Oct 2020	59	112	53
B.1.617.2	VOC	Oct 2020	57	112	55
B.1.526	VOI	Nov 2020	23	65	42
B.1.525	VOI	Dec 2020	26	45	19
P.3	VOI	Jan 2021	31	45	14
B.1.427	VOI	March 2020	53	291	238
B.1.429	VOI	March 2020	59	247	188
B.1.621	VOI	Jan 2021	21	210	189
P.2	VOI	April 2020	121	184	63
B.1.620	VUM	Nov 2020	108	143	35
B.1.1.529	VOC	Nov 2021	15	17	2
C.37	VUM	Dec 2020	87	181	94
C.1.2	VUM	May 2021	45	90	45
AV.1	VUM	March 2021	42	55	13
AT.1	VUM	Jan 2021	101	198	97
B.1.630	VUM	March 2021	93	201	108
B.1.466.2	VUM	Nov 2020	99	151	52

Pango Lineage: lineage according to the Pango nomenclature. VOC/VOI/VUM: status as VOC, VOI or VUM. Time first detected: time of isolation of the first genome associated with the variant. Days from first detection to HG: days intercurring from the isolation of the first genome to the appearance of a corresponding HG. Days from detection to VOC-VOI-VUM: days intercurring from the isolation of the first genome associated with a variant to the acknowledgment of that variant as VOC/VOI/VUM by WHO. Days from HG designation to VOC/VOI/VUM: days intercurring from the designation of an HG by HaploCoV to the acknowledgment of the status of a VOC/VOI/VUM according to WHO.

**Table 3:** Prioritization of VOC/VOI/VUM lineages.

Ancestral lineage (Pango)	Date first genome (GISAID)	Score above threshold	
		From	To
B.1.1.7	2020-09-03	Oct 2020	Feb 2022
AT.1	2021-01-18	Mar 2021	Feb 2022
AV.1	2021-03-12	Apr 2021	–
B.1.1.318	2021-01-07	Mar 2021	Mar 2022
B.1.1.519	2020-08-28	No	No
B.1.1.523	2021-01-02	Feb 2021	Dec 2021
B.1.1.529	2021-11-23	Dec 2021	–
B.1.214.2	2020-11-22	Jan 2021	Dec 2021
B.1.351	2020-05-01	Dec 2020	Dec 2021
B.1.427	2020-03-11	Jun 2020	Dec 2020
B.1.429	2020-03-01	May 2020	Dec 2020
B.1.466.2	2020-11-06	No	No
B.1.525	2020-12-15	Jan 2021	–
B.1.526	2020-11-03	Dec 2020	Jan 2021
B.1.617.1	2020-10-01	Oct 2020	Dec 2021
B.1.617.2	2020-10-05	Oct 2020	–
B.1.619	2020-05-08	Jun 2020	Jan 2022
B.1.620	2020-05-09	Jun 2020	Jan 2022
B.1.621	2021-01-15	Feb 2021	Jan 2022
B.1.630	2021-03-17	Apr 2021	Feb 2022
B.1.640	2021-09-24	Oct 2021	–
C.1.2	2021-05-11	Jun 2021	–
C.36.3	2021-01-04	Feb 2021	Feb 2022
C.37	2020-12-22	Jan 2021	Jan 2022
P.1	2020-11-01	Jan 2021	Feb 2022
P.2	2020-04-13	May 2020	Sept 2020
P.3	2021-01-08	No	No
R.1	2020-05-17	No	No

Ancestral Lineage (Pango): ancestral lineage according to the Pango nomenclature. Date first genome (GISAID): collection date of the first genomic sequence assigned to the variant. Score above threshold: interval of time a variant was scored above the threshold for prioritization. From = start date. End = end date (dash = no end date, i.e score above the threshold at the time of the execution of the analyses). No: the designation was never associated with a score surpassing the minimum threshold.

#### Reproducibility and stability of the results

A bootstrap analysis was applied to evaluate the consistency and reproducibility of the HGs established by HaploCoV. Twelve distinct time intervals of roughly 30 days, corresponding to the 12 months spanning from December 2020 to December 2021, were considered. At every interval novel genome sequences were incorporated, based on the deposition date as indicated in the GISAID EpiCoV database, and the classification was augmented incrementally. A total of 1000 bootstrap replications were performed at every iteration and the following metrics were collected to measure the stability and reproducibility of our approach: average phenetic distance (dissimilarity) between pairs of closest (most similar) HGs formed at distinct bootstrap iterations; number of genomes inconsistently assigned to distinct HGs at matched time-points; variability in the total number of genomes assigned to the closest HG at the same time point, at different bootstrap replicates.

**Fig. 3:**
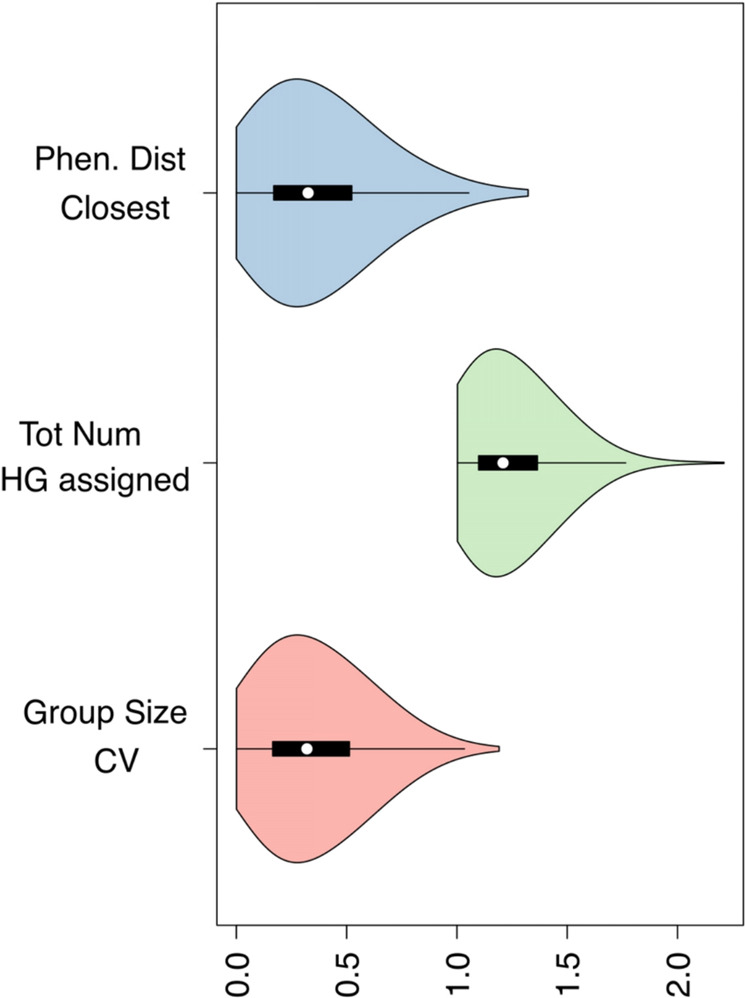
Stability and reproducibility metrics. Phen. Dist. Closest HG pairs**:** phenetic distance between closest pairs of HGs formed at distinct bootstrap iterations. This metric measures the similarity between matched HGs formed at different iterations. A value of 0 indicates completely identical HGs (defined by an identical set of genomic variants). Tot. num. HG assignment per genome**:** distribution of the total number of distinct HGs to which a genomic sequence was assigned at distinct bootstrap iterations. A value of 1 indicates perfect reproducibility (i.e., the genome was consistently assigned to the same HGs at every iteration). HG size CV**:** coefficient of variation (CV) of the size (total number of genomes assigned) to matched HGs formed at distinct bootstrap iterations. A value of 0 indicates no variability in the size of matched HGs (i.e., the same number of genomes is assigned to matched HGs at distinct iterations). Distributions are represented in the form of a violin plot. In the boxplots, white dots denote median values; boxes extend from the 25th to the 75th percentile; vertical extending lines denote adjacent values (i.e., the most extreme values within 1.5 interquartile range of the 25th and 75th percentile of each group). The source data behind this figure is reported in Supplementary Data 19.

As outlined in Fig. 3, our framework consistently delineated nearly-identical HGs defined by the same sets of genomic variants across independent iterations. Indeed, pairs of closest/equivalent HGs formed at distinct iterations showed a phenetic distance close to 0 (Phen. Dist. Closest), indicating that they were defined by the same set of genomic variants, and were identical. Assignments of genomes to HGs were highly reproducible and, at every bootstrap replicate, genomic sequences were consistently assigned to the same HGs (Tot Num assigned: average number of HGs to which a genome was assigned at distinct iterations close to 1). Finally, HGs formed by our approach at different bootstrap replicates were nearly identical in size (the coefficient of variation of HG size, Group size CV is close to 0), indicating a highly consistent composition with very limited variability. All in all, these results indicated that the approach applied by HaploCoV to delineate HGs is highly reproducible and robust.

### Regional genomic diversity in large SARS-CoV-2 lineages

Applied to the 11.5 Million SARS-CoV-2 genomic sequences available in the GISAID EpiCoV database on 10th June 2022 (Supplementary Data 6), our HapoCoV formed a total of 1571 distinct HGs in an unsupervised manner, compared with the 1720 lineages defined by the Pango nomenclature. Interestingly, 913 Pango lineages were supported by <100 genomic sequences and/or were not defined by a unique combination of two or more high frequency genomic variants. Accordingly these lineages were not identified as distinct designations by HaploCoV. Conversely the large majority of Pango lineages that reached a widespread circulation and incorporated a large number of genomes corresponded with two or more HGs in HaploCoV. For example the B.1.1.7 lineage, which included more than 1.1 M of genome sequences according to the Pango nomenclature, was partitioned in 209 HGs by HaploCoV; the AY.4 lineage (865k genomes) in 147 HGs; the BA.1 lineage (511k genomes) in 44 HGs and the BA.2 lineage (1.1 M genomes) in 59 HGs. Importantly, the additional designation/groups formed by HaploCoV in these lineages were supported/coherent with phylogenetic analyses and, as illustrated in Supplementary Fig. S5 (A–C), HGs comprising 1500 or more sequences tended to form distinct and well supported clusters/clades in the phylogeny of the isolates. Taken all together, and also in the light of the high levels of reproducibility described above, we believe that these observations provide convincing evidence concerning the validity of the approach implemented by our method.

A highly significant positive correlation (*p* value < 1e−32; *R*^2^ = 0.59) was observed between the total number of additional HGs formed within a Pango lineage and the total number of genomes assigned to that lineage. Consistent with this observation while a total of 9, 242 and 98 Pango lineages were incorporated within the Alpha, Delta and Omicron VOC respectively, equivalent figures account for 213, 554, and 184 HGs in HaploCoV indicating an almost threefold increase.

To demonstrate the integration of our novel software framework with current nomenclature standards, we applied HaploCoV to extend/complement the Pango nomenclature. A total of 887 novel additional designations were formed within Pango (Pango + lineages hereafter), raising the total combined number to 2607 (Supplementary Data 7). Notably, 84% (747 out of 887) of the newly formed Pango+ lineages and HGs identified in an unsupervised manner by HaploCoV were defined by identical sets of high frequency genomic variants, suggesting a high degree of reproducibility.

Consistent with our previous observations, a highly significant positive correlation (*p* value < 1e−32; *R*^2^ = 0.61) was observed between the total number of genomic sequences assigned to a Pango lineage and the number of Pango+ lineages formed within that lineage. A large number of Pango+ lineages were delineated within the Alpha, Delta and Omicron VOC: 167, 350 and 289 respectively (806 out of 887).

Cumulatively, Pango+ lineages incorporated a total of 557 high frequency genomic variants (Frequency > 1%) that were not associated with any lineage defined by the Pango nomenclature (Supplementary Data 8). Among these genomic variants, 82 had a widespread geographic distribution (Frequency > 1% in more than 2 macro-geographic regions and more than 10 countries), while the large majority (475) showed a more restricted geographic circulation (Frequency > 1% in only 1 macro area and <5 countries). Consistent with these observations (Supplementary Data 9) a large proportion (329/887 = 37.09%) of the Pango+ lineages were associated with a local geographic distribution, and were associated with 10 or less distinct countries.

Based on the analysis of the first 50 isolates, as reported in GISAID EpiCoV (Supplementary Data 10), 668 Pango+ lineages could be tentatively assigned to a country of origin (50% or more of early isolates associated with a single country). A highly significant positive correlation was observed between the total number of distinct Pango+ lineages assigned to a country of origin and the total number of genomic sequences deposited in the GISAID EpiCoV database from that country (*p* value < 2e−16; *R*^2^ = 0.51), indicating that also the identification of a country of origin is potentially influenced by sampling biases. Indeed we notice that, apart from the countries/geographic regions of first isolation, Pango+ lineages corresponding with VOCs/VOIs/VUMs were preferentially associated with countries that contributed the highest numbers of viral genome sequences to the GISAID EpiCoV database. For example, Pango+ lineages corresponding with B.1.526 and B.1.429 were prevalent in North America, while the majority of Pango+ lineages associated with the Omicron and Delta variants were detected either in the USA or UK. This notwithstanding, we also found a limited number of Pango+ lineages with a more widespread geographic distribution, which likely reflect independent introductions in distinct countries; these include Pango+ lineages associated with the Gamma and Beta VOCs (B.1.351.N1 (Beta); P.1.N2 (Gamma)).

Globally, Pango+ lineages associated with VOC/VOI/VUM incorporated in the excess of 3.64 M genomic sequences corresponding to 38.45% of the 9.56 M genomes cumulatively assigned to VOC variants by Pango (Supplementary Data 11). While 806 over 887 of Pango+ lineages were associated with Omicron, Alpha and Delta, a more limited number of additional groups were also delineated within variants that did not become highly prevalent worldwide; these include: the Beta and Gamma VOC (9 and 3 Pango+ lineages respectively), the Epsilon (4 Pango+) and Iota (3 Pango+) VOI and the R.1 VUM (1 Pango+) (see Supplementary Data 11).

Taken together, these results emphasize the existence of local viral variants not explicitly addressed by current nomenclature standards, and suggest potential implications for the fine-scale mapping of routes of transmission of viral variants across different areas and countries.

### Epidemiologically relevant variants have characteristic mutational signatures

Applied to the Pango nomenclature, our approach delineated a total of 887 novel candidate designations (Pango+ lineages). Systematic manual evaluation of such a large number of novel candidate lineages for their incorporation in Pango would require long turn-around times. In this respect the development of automated systems for the scoring/prioritization of novel emerging variants would be highly advisable. In the case of SARS-CoV-2, such a method could benefit from the large body of data that has accumulated over the previous 2.5 years.

**Fig. 4:**
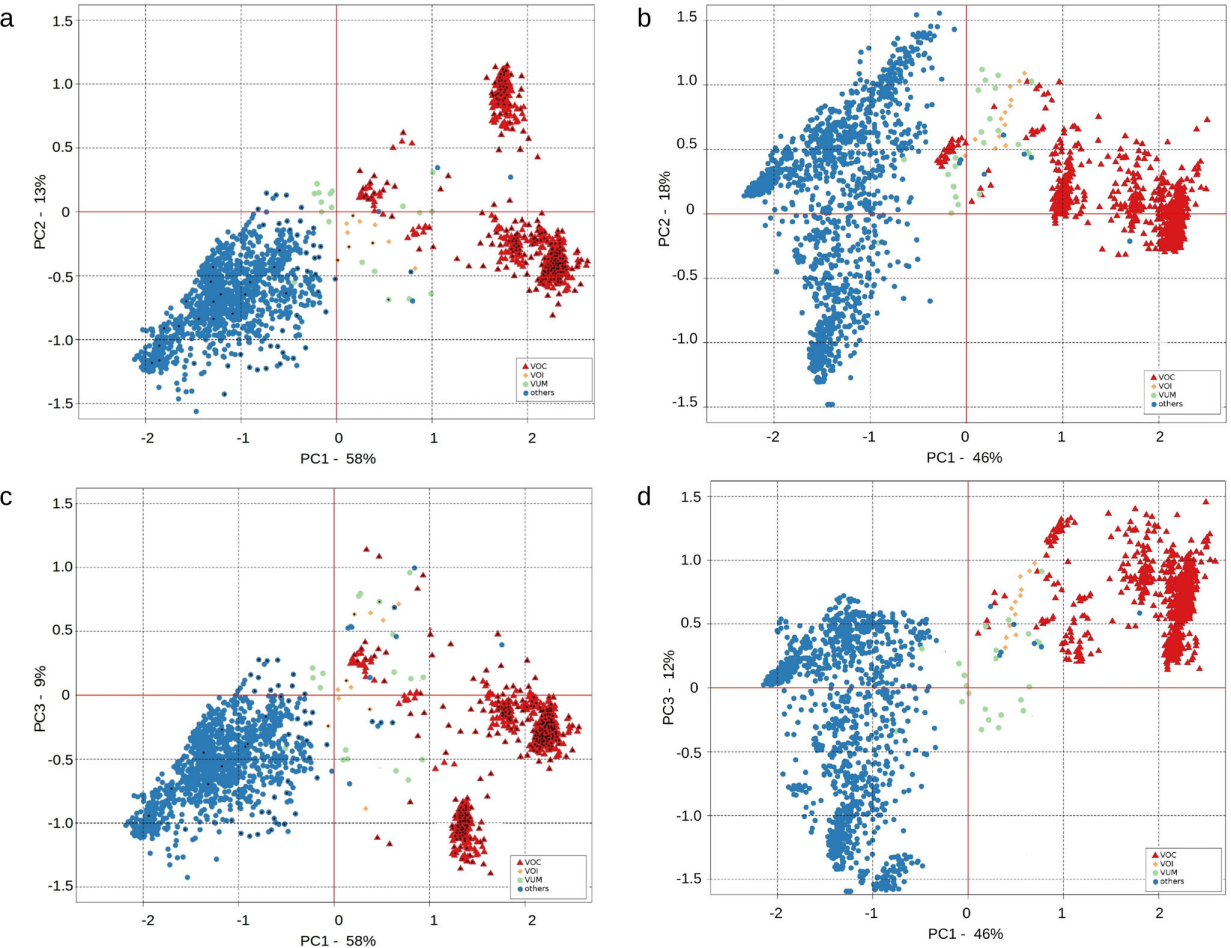
PCA representation of 113 descriptive features for the 2607 Pango/Pango+ lineages and the 1571 HGs. Distinct colors are used to represent VOC (red), VOI (yellow), VUM (green) and other variants (blue). **a** Pango/Pango+ Lineages, PC1 and PC2. Pango+ lineages are. **b** HGs, PC1 and PC2. **c** Pango/Pango+ Lineages, PC1 and PC3. **d** HGs, PC1 and PC3. Pango+ lineages are marked by an overlaid black dot. The 113 descriptive features are listed in Supplementary Data 12.

A set of 113 descriptive features (see Supplementary Data 12) were recorded for all of the 2607 Pango/Pango+ lineages and the 1571 distinct HGs delineated by HaploCoV. These features included: total number of defining genomic variants (i.e., genomic variants with a frequency of 50% or above within a specific HG or Pango lineage); total number of synonymous and non-synonymous variants; measures of global genomic diversity; counts of sites predicted to be under positive and/or negative selection; number of predicted/validated viral epitopes recognized by IgG and IgM and associated with non-synonymous substitutions; counts of local/global high frequency alleles (at different frequency thresholds) and accessibility in the secondary structure of the viral genome. Features were scaled using unity based normalization, and dimensionality reduction was applied to identify potential patterns of similarity/dissimilarity between SARS-CoV-2 variants. PCA plots in Fig. 4a–c and Fig. 4b–d show a good stratification of distinct types of SARS-CoV-2 variants (VOC/VOI/VUM and non VOC/VOI/VUM variants, referred to as “other variants” here onward). Importantly the large majority of VOCs/VOIs/VUMs clustered together both in the analysis of Pango/Pango+ lineages and HGs, with the first component of the PCA (PC1, *x*-axis) corresponding to the separation of VOCs/VOIs/VUMs from other viral variants. Moreover, when PC2 and PC3 (panels 4a–c versus 4b–d) were considered, most VOI and VUM variants were placed on the edge between the other variants and most/all VOCs for both Pango/Pango+ lineages and HGs. These results clearly indicate that the simple metrics we adopted can reliably identify compact and discrete groups of SARS-CoV-2 variants that incorporate all current VOCs/VOIs/VUMs and suggests that our approach could be effectively applied to facilitate the identification of potential VOCs and VOIs emerging in the future.

A total of 58 features showed a statistically significant difference in their distribution in VOC/VOI/VUM lineages compared to other variants (*P* value < 0.01 in Supplementary Data 12). In general, VOCs, VOIs and VUMs displayed an overall increase in genomic diversity, including: a higher number of defining genomic variants (here defined as genomic shared by at least 50% of the genomic sequences), an increased proportion of incompletely fixed genomic and a higher mutational load at viral epitopes.

**Fig. 5:**
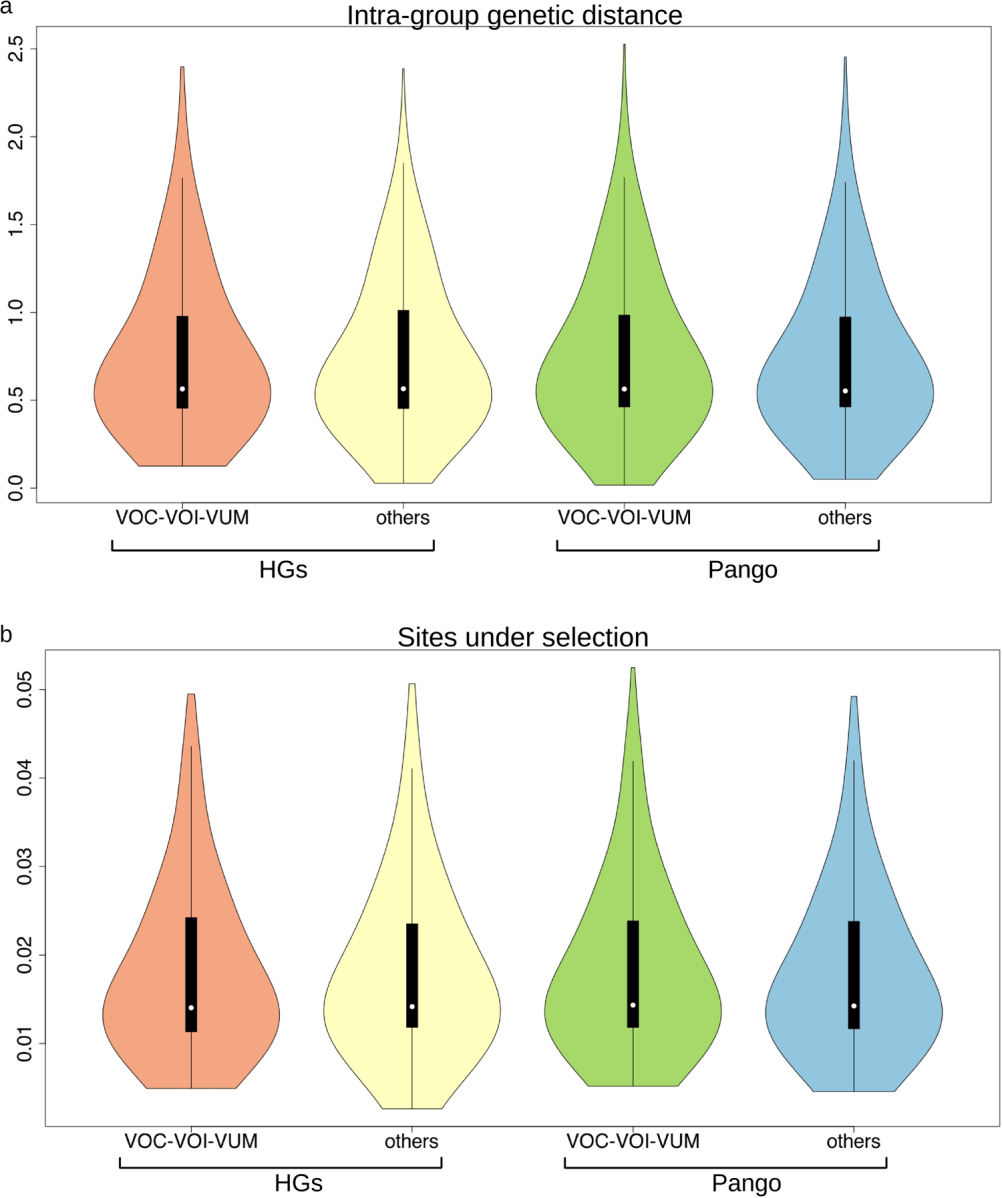
Distribution of the intra-group genetic distance and sites under selection in VOCs/VOIs/VUMs compared to other variants. **a** Distribution of genetic distance, computed as the total number of distinct polymorphic sites between closest pairs of genomes assigned to Pango/Pango+ lineages and HGs, separately for VOC/VOI/VUM and for non VOC/VOI/VUM variants (indicated as others). Distances are indicated on the *Y*-axis. **b** Proportion of non-defining genomic variants predicted to be under selection (according to Hyphy) in Pango/Pango+ lineages and HGs separately for VOCs/VOIs/VUMs and for all remaining variants (indicated as others). Ratios are indicated on the *Y*-axis. Distributions are represented in the form of a violin plot. In the boxplots, white dots denote median values; boxes extend from the 25th to the 75th percentile; vertical extending lines denote adjacent values (i.e., the most extreme values within 1.5 interquartile range of the 25th and 75th percentile of each group). The source data behind this figure is reported in Supplementary Data 20.

Notwithstanding a higher number of defining genomic variants (27.9 compared to 10.6 on average), VOC/VOI/VUM did not display an increased intra-group variability compared with other viral variants not flagged by WHO (Fig. 5a). Consistent with this observation, when defining high frequency genomic variants were excluded, an overall similar proportion and distribution of sites potentially associated with positive selection, as determined by Hyphy^22^ (Supplementary Data 13), was observed in VOC/VOI/VUM and other variants of SARS-CoV-2 (Fig. 5b). Taken together these findings suggest that the increased diversity associated with the most successful/widespread variants of SARS-CoV-2 should not reflect an alteration of the evolutionary dynamics of the virus per se; but is rather compatible with the rapid episodic emergence and fixation of viral haplotypes with increased fitness, followed by accumulation of passenger mutations.

Three genomic regions were found to show an excess of genomic variability in SARS-CoV-2 VOC/VOI/VUM: the S glycoprotein (adjusted Fisher *p* value = 1e−15); the N protein (adjusted Fisher *p* value = 1e−13); and s2m (adjusted Fisher *p* value = 1e−4), a 43 bp secondary structure element of unknown function which is found in several families of single-stranded RNA viruses^23^. Cumulatively, 88 genomic variants displayed a significant over-representation (adjusted Hypergeometric *p* value ≤ 0.05) in VOC/VOM/VUI compared to other variants of SARS-CoV-2. Among these 88 genomic variants, 82 fall in protein-coding genes (65 non synonymous substitutions, 7 inframe deletions and 10 synonymous substitutions) and 30 are predicted to be under positive selection (adjusted *P* value hypergeometric ≤ 1e−14) according to Hyphy; strikingly these last include all of the most relevant mutations associated with VOCs and VOIs in the S glycoprotein, including E484K, N50Y, P681H/R and L452R, along with several additional genomic variants, whose relevance has been already confirmed by in vitro studies^24–26^ (see for example G142D, K417N, G496N, T478K in Supplementary Data 14).

#### A simple approach for the prioritization of emerging SARS-CoV-2 variants

To derive a scoring system for the prioritization of SARS-CoV-2 variants, the 58 genomic features associated with statistically significant differences in their distribution of values in VOC/VOI/VUM compared to other variants of SARS-CoV-2 were ranked based on levels of statistical significance (*p* values). Subsequently linear scores were computed by progressive aggregation of the N top ranking features (with N ranging from 5 to 58). A total of 54 distinct scoring systems were derived. Distribution of scores associated with VOC/VOI/VUM were compared with those derived for the other variants of the virus. Highly statistically significant differences were observed under every scoring system (Supplementary Data 15), with levels of significance ranging from 1.14e−11 (scoring system #38) to 7.42e−15 (scoring system #39), according to a Wilcoxon rank sum test.

To identify the most effective system and metrics for the prioritization of SARS-CoV-2 variants, a time course analysis was performed on an interval of time ranging from Jan 2020 to May 2022. Recombinant lineages in the Pango nomenclature were excluded from these analyses based on the consideration that the large majority of these lineages (31/32) derived from the recombination of one or more VOC/VOI. At every consecutive month newly formed lineages in the Pango nomenclature were incrementally added, functional annotations of genomic features were updated, and prioritization scores were re-computed accordingly, for every lineage.

The ideal threshold for the prioritization of VOC/VOI/VUM under each scoring system was determined empirically, by counting the number of VOC/VOI/VUM and other variants with a score above a given threshold value. The following metrics were computed: total number of VOC/VOI/VUM lineages/variants with a score above the threshold for prioritization (true positives); total number of other variants with a score above the threshold.

Evaluation of the results was undertaken both by aggregating lineages at the level of variants according to the definition by WHO, and both by considering each single lineage as a distinct entity.

Under these criteria, the model based on the 30 best top ranking features, which correctly prioritized 98.51% of VOC/VOI/VUM Pango lineages, and at the same time resulted in the the prioritization of the lowest number of other variants/lineages not associated with VOC, VOI or VUM (3.36% corresponding to 42 lineages) was considered the most accurate (scoring system #26 in Supplementary Data 15).

A complete list of the Pango lineages reaching a score above the empirically established threshold for the prioritization is reported in Table 3 and Supplementary Data 16 for the VOC/VOI/VUM and the other variants, respectively. Notably, the large majority of VOC/VOI/VUM variants (86.2%) were prioritized in an unsupervised manner within a maximum of 2 months from the availability of the first genomic sequence (Table 3).

A total of 4 VOI/VUM variants were not correctly prioritized by our system. Interestingly, we observe that these variants carry a reduced number of defining non-synonymous substitutions in the Spike glycoprotein (3–7, including the ubiquitous D614G substitution) and reached a prevalence of 1% or above only in a single country: B.1.1.519, Mexico 10%; B.1.466.2, Indonesia, 8%; R.1, Sierra Leone, 30%; P.3 Philippines: 3%.

Conversely our approach identified a total of 42 lineages that were not previously flagged as VOC/VOI/VUM by the WHO (Supplementary Data 16), also in agreement with the results of the PCA analyses of the 113 descriptive genomic features (see other variants blue points clustering with VOC/VOI/VUM points in Fig. 4). Among these 42 lineages, 15 were subjected to monitoring by regional health authorities and/or were reported as “escape variants” in the literature^27–36^; 13 reached a prevalence of 1% or above in at least one or more countries, and 3 reached a global prevalence of 5% or above worldwide (B.1.177, B.1.160 and B.1.2). Two further lineages were reported to be associated with zoonotic transmission of SARS-CoV-2 to non human hosts^37,38^. Finally a total of 8 lineages were associated with a score greater than the cut-off for prioritization only in the interval of time that pre-dates the emergence of the Alpha and Beta VOC (Supplementary Data 16). Interestingly, 3 of these 8 lineages: B.1.177, B.1.160 and B.1.2 were also associated with a high prevalence (5% or above) worldwide^39^, an observation that might be consistent with selective advantages.

## Discussion

Genomic surveillance of SARS-CoV-2 requires rapid and effective approaches for the identification of novel strains/lineages of the virus as they emerge, coupled with accurate models for flagging variants of potential concern and identifying those that should be subjected to more careful monitoring. Ideally these approaches should be completely automated, to reduce turn-around times and potential biases and should be based on deterministic, human interpretable rules to facilitate expert manual revision and curation of the results.

Here we present a novel highly flexible approach for the exploration of genomic diversity in SARS-CoV-2, which extends the range of application of our original strategy proposed in Chiara et al.^16^ and can be integrated with any existing nomenclature/classification system, to enable a rapid and systematic identification of novel emerging variants/strains, including current and future VOC, VOI and VUM.

We demonstrate that our approach can correctly delineate one or more related designations for all current and past VOC, VOI and VUM as defined by international health authorities. With an average 70 days between the availability of the first genomic sequence of a corresponding group, this timeframe is completely compatible with that required for the identification of variants of epidemiological interest by International Health authorities (Table 2).

In addition, we observe that a substantial proportion of high frequency genomic variants associated with various countries and macro-geographic areas are not captured by current nomenclature standards, a consideration that might have important implications for the accurate and rapid tracking of novel, emerging country/region specific variants of the virus.

For example, we often observe that current nomenclature standards do not capture large groups of genomic sequences characterized by specific combinations of high frequency genomic variants and associated with a limited number of geographic locations. This suggests the importance of the incorporation of local frequency estimates for the timely identification of new viral haplotypes. Importantly, we observe that these considerations apply in particular to large and rapidly-expanding SARS-CoV-2 lineages (see Supplementary Data 11) and to several VOCs/VOIs/VUMs.

Finally, we illustrate a simple scoring system, based on genomic features, for the identification/prioritization of SARS-CoV-2 lineages/haplogroups of potential epidemiological relevance. By performing retrospective analyses, we demonstrate that our approach can identify the large majority of lineages of SARS-CoV-2 recognized as VOI/VUM/VOC in a timely manner, and provides a reliable and automatic method for the prioritization of SARS-CoV-2 variants which might represent a source of concern (Table 3).

Due to the profound mechanistic importance of the Spike glycoprotein, and thanks to our detailed knowledge of its molecular interactions, SARS-CoV-2 nomenclature standards have tended to focus on variants in the *S* gene^24–26^, whose potential biological relevance can often be rapidly flagged through expert intervention. The weights assigned to *S* related features in our approach for SARS-CoV-2 variant prioritization confirms the epidemiological importance of *S* gene evolution, but the relevance of other features highlights an additional set of genomic variants specifically enriched in VOCs/VOIs/VUMs. For example, we notice an increased genomic diversity in the *N* nucleocapsid protein gene. Although the primary functions of the *N* gene are the binding and packing of the viral RNA genome, *N* is a highly immunogenic protein and is expressed at extremely high levels during infection^40^. Interestingly, the observation that several non-synonymous substitutions in *N* found in VOC/VOI/VUM are under selection (as predicted by Hyphy) and are associated with predicted viral epitopes might suggest a link with evasion of anti-N neutralizing antibodies and indicates a need for further investigation.

We observe that VOC/VOI/VUM are associated also with an excess of variability at the s2m locus compared with other variants. According to recent studies, s2m represents one of the most variable genomic elements in the genome of SARS-CoV-2^16^. Since the reference genome assembly carries a nucleotide substitution in s2m at a highly conserved and structurally important position (T at pos. 29,758), with possible impacts on its structural stability, it was speculated that the observed excess in genomic diversity could be compatible with either loss of functional constraints and/or diversifying selection^16^. Interestingly, we observe that the G to T substitution at 29,742, which is a hallmark of the Delta VOC (and related lineages), is associated with a considerable increase in the predicted secondary structure stability of s2m. With a cumulative frequency of 20%, this substitution is one of the most frequent genomic variant in s2m, and is also associated with 36 distinct (non Delta) variants, showing a high prevalence (10% and 7% in central and southern Asia, respectively) even prior to the emergence of Delta. At the same time, we observe that more than 1 M of genomic sequences associated with the BA.2, BA.3 and BA.5 lineages of the Omicron variant, corresponding to 9.5% of the total number of SARS-CoV-2 genome sequences analyzed in this study, harbor a deletion of 27 bp at the s2m locus, suggesting a partial or complete loss of function. These observations are consistent with the hypothesis that genomic variants contributing to increased s2m structural stability may be positively selected, but this secondary structure element might be also subjected to general loss of constraints or diversifying selection, at least in the Omicron variants.

Notwithstanding these interesting observations and the good results demonstrated by our approach in the identification of SARS-CoV-2 VOC/VOI/VUM it should be underlined that the simple linear scoring system used by HaploCoV, can not capture synergistic interactions and/or complex epistatic effects between different genomic variants and can not be used to address/interpret complex evolutionary patterns. In lieu of these considerations, the development of more complex and specialized machine learning based approaches, which could also take into account data concerning protein conformational structure and dynamics is probably required to translate some of the observations reported in this work into mechanistic insights and/or testable biological hypotheses.

**Fig. 6:**
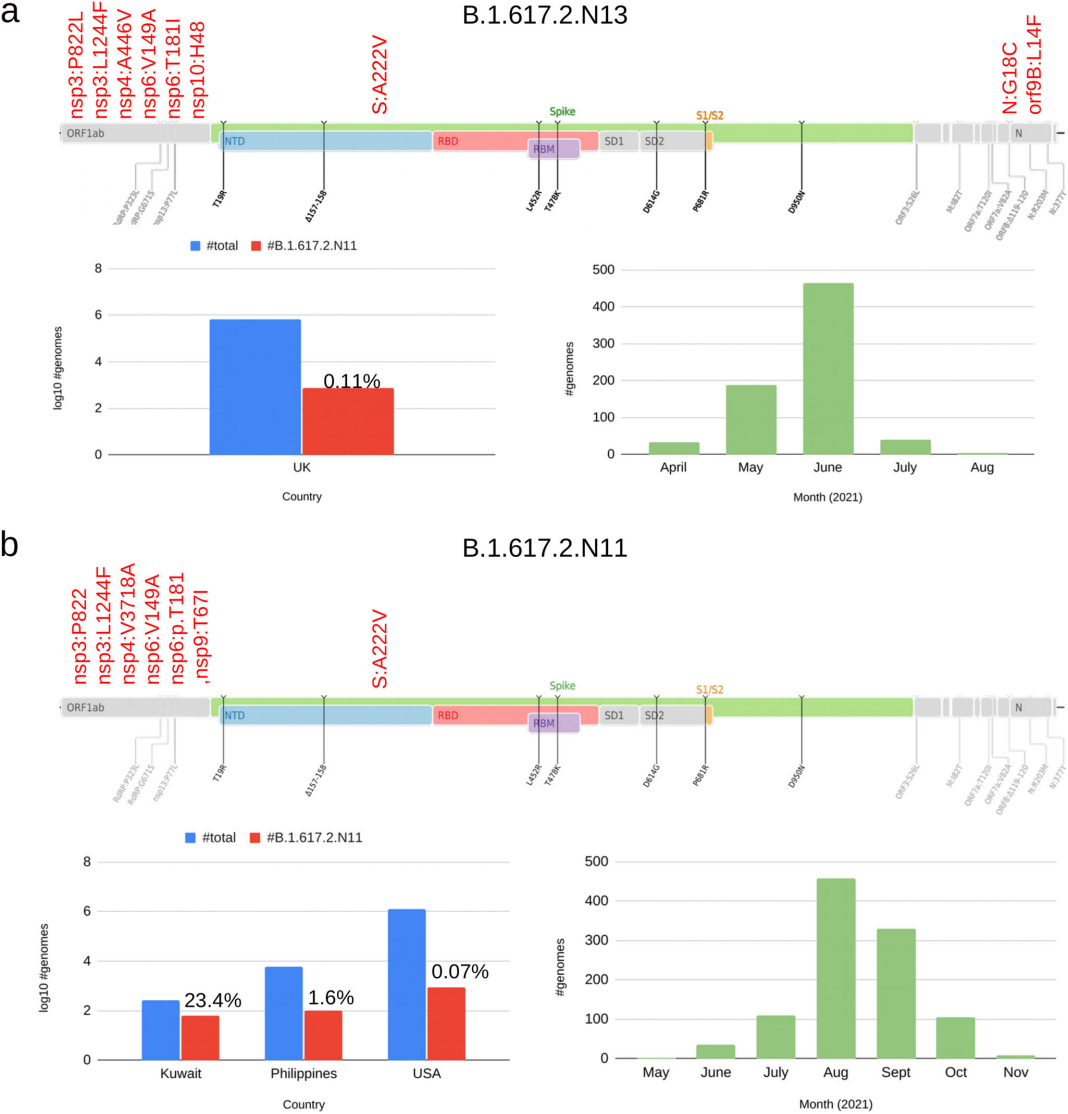
Interesting Pango+ lineages derivative of Delta VOC. **a** Defining genomic variants and prevalence of the B.1.617.2.N13 Pango+ lineage. Red: novel genomic variants specific to the Pango+ lineage; black: genomic variants characteristic of Delta in the Spike glycoprotein (S); gray: genomic variants characteristic of Delta outside the S gene. Barplots represent the total number of sequences and estimated prevalence of B.1.617.2.N13 in countries from which more than 50 genome sequences were recovered (log10 scale) and total number of genome sequences assigned to B.1.617.2.N13 from April 2021 to August 2021. **b** Defining genomic variants and prevalence of B.1.617.2.N11 Pango+ lineage. Red: novel genomic variants specific to the Pango+ lineage; black: genomic variants characteristic of Delta in the Spike glycoprotein (S); gray: genomic variants characteristic of Delta outside the S gene. Barplots represent the total number of sequences and estimated prevalence of B.1.617.2.N13 in countries from which more than 50 genome sequences were recovered (log10 scale) and total number of genome sequences assigned to B.1.617.2.N13 from May 2021 to November 2021. Annotation of protein-coding genes and protein domain according to the Uniprot annotation (UP000464024) of the reference SARS-CoV-2 genome (NC_045512). The source data behind this figure is reported in Supplementary Data 21.

Over 11.5 M SARS-CoV-2 genome sequences have been investigated in the present study. The analysis, interpretation and classification of this unprecedented catalog of genomic variation represents a tremendous challenge for modern genomic science. While phylogenetic analyses of regional viral isolates facilitate fine-scale mapping of single chains of contagion and the tracking of routes of transmission across different regions and countries, they may not contribute as explicitly to the rapid identification of novel “epidemiologically relevant” strains. The approach proposed here occupies the middle-ground between these two contrasting necessities, wherein broad-scale analysis could be applied for the systematic identification of groups of variants circulating in a geographic area or country, and subsequently viral variants of potential epidemiological interest could be identified, either through direct evaluation of the linear score proposed here, or by comparing scores of novel designations with those of the corresponding ancestral states. A simple utility to execute this last type of analysis is already included in our software framework. For example, we note that only 7% (61/887, Supplementary Data 17) of the Pango+ lineages formed by our approach show an increase in their prioritization score of 2 or greater compared to the corresponding ancestral lineage. Such a limited number of novel designations could be easily integrated within any nomenclature system. Interestingly, all the Pango+ lineages associated with increased prioritization scores derive from VOCs (Gamma, Delta and Alpha), carry an excess of genomic variants (from 4 to 15) compared with their respective ancestral lineage and are prevalently localized in a single country. Among these, there are two potential derivatives of the Delta variant, B.1.617.2.N11 and B.1.617.2.N13, which carry an additional set of 7 and 9 non synonymous substitutions, respectively compared with the ancestral Delta lineage, and reached a moderate prevalence in the USA and in the UK (Fig. 6) prior to the emergence of the Omicron variant. Our attention was also drawn by the relatively large number (38) of potential derivatives of the Alpha variant, since the Pango+ designations with an increased prioritization score within Alpha collectively include more than 92k genomes. Interestingly, several of these Pango+ lineages (15 out of 38) are partly defined by additional genomic variants in the *ORF8* gene which is thought to have been inactivated in the ancestor of Alpha^41,42^.

Although optimal settings for the application of HaploCoV to the analysis SARS-CoV-2 genomic data were defined in this study, we underline that the approach implemented by our method is highly flexible and enables the application of different settings and thresholds. For example, the threshold for the delineation of novel groups/designations can be arbitrarily adjusted by the users according to their specific needs: if the objective of the analysis is to track and evaluate each distinct high frequency genomic variant that has been fixed in the genome of SARS-CoV-2, the threshold value can be reduced to 1; conversely users aiming to identify and flag only groups of genomic sequences that differ by a large number of genomic markers with regards to parental designations can increase the threshold value accordingly. Examples on how to modify this and many other settings are provided in the manual of HaploCoV.

In addition, while by default HaploCoV employs pre-computed collections of high frequency genomic variants to derive novel potential designations; users can configure our tool to derive sets of high frequency genomic variants based on any arbitrary selection of genomic sequences of choice. For example, by considering only genomes associated with a specific country or region; or genomes isolated from a user-defined interval of time. In principle, this approach enables the identification of novel clusters of viral genomes/lineages defined by any combination and any number of genomic variants. In addition, also the threshold for the minimum number of viral isolates required to form a new designation in HaploCoV, can be adjusted to fit/meet specific requirements (i.e., small groups of highly variable genomes or larger groups defined by a limited number of genomic variants).

While the application of different sets of parameters and criteria could potentially lead to the formation of large numbers of additional candidate designations, prioritization strategies based on the HaploCoV score—as those outlined above—provide an effective means to reduce the breadth of the results and identify novel variants with VOC/VOI/VUM-like genomic features, which could be worth including in the target nomenclature system. HaploCoV already incorporates ad-hoc utilities for the post-processing of the results, to generate informative reports and facilitate the identification and profiling of interesting/relevant viral variants that display a widespread circulation.

For example, a fine grained search for highly variable clusters of viral genomes, defined by 6 or more additional genomic variants with respect to their parental strain and supported by 5 or more isolates, identified a total of 227 novel potential additional designation in the Pango nomenclature when sequences, as available from May 1st 2022 to June 30th 2022 were considered. Among the novel designations only 9 displayed a marked increase in their HaploCoV prioritization score (Supplementary Data 18); notably this shortlist included BA.2.N39, a designation formed by HaploCoV in an unsupervised manner, which corresponded to BA.2.75, a Pango lineage which was designed on June 26th 2022, and was put under scrutiny by the WHO^4^.

In conclusion we have proposed and validated a fully automated “epidemiogenomic” approach that can be used in isolation, or alongside the Pango nomenclature, to provide rapid, flexible and granular tracking of SARS-CoV-2 genomic diversity and to highlight emerging epidemiologically relevant strains in a timely and accurate manner.

## Methods

### Data retrieval

The complete collection of SARS-CoV-2 genomic sequences and associated metadata (including lineage designation according to the Pango nomenclature) was retrieved directly from gisaid.org on June 10th 2022. Data concerning the incidence and total number of cases of COVID-19 were downloaded from the WHO dashboard, at https://covid19.who.int/ on the same day.

### High quality genomes

Custom Perl scripts were used to infer the size of each genomic assembly and the number of uncalled bases/gaps (denoted by N in the genomic sequence). High quality genomic assemblies were initially defined, according to the criteria outlined in Chiara et al.^16^, as genome sequences longer than 29,850 nt and including less than 150 ambiguous sites. These criteria were revised in order to prevent possible biases in the computation of allele frequency distributions, since systematic differences were observed when comparing genome assemblies derived from different countries, and due to the frequent incompleteness of the 5′- and/or 3′-UTR (having a length of 229 and 265 nt, respectively). Therefore, sequences with <350 ambiguous sites, and with <350 bases missing at either the 3′ or 5′ end of the genome (i.e., longer than 29,550 nt) were considered of high quality. Only sequences satisfying these criteria were considered for the computation of genomic variant frequencies and the delineation of groups of SARS-CoV-2 genomes.

### Haplogroups and functional annotation

Bioinformatics analyses for the delineation of novel HGs and the assignment of genomic sequences to HGs were performed according to the methods described in Chiara et al.^16^.

Briefly, SARS-CoV-2 genomes were aligned to the 29,903 nt-long reference assembly of SARS-CoV-2 by means of the nucmer program^43^. Genetic variants were identified by nucmer show-snp. Frequency of genomic variants were determined by the computeAF.pl utility in HaploCoV. Genomic sequences were assigned to a macro-geographic region based on the country of origin of the genome as reported in the GISAID EpiCoV database. Correspondence between countries and macro-geographic regions is reported in Supplementary Data 3. Functional annotation of genomic variants was performed by CorGAT^44^ using the annotation tracks listed in Supplementary Data 12^45–47^. Designation of VOC, VOI and VUMs lineages was in accordance with the WHO report of June 10th 2022^4^.

Haplogroups (HGs) were established by agglomerative hierarchical clustering of phenetic profiles of presence/absence of high frequency genomic variants, as implemented by the augmentClusters.pl utility in HaploCoV. The–dist and–size parameters were set to 2 and 100 respectively.

Existing nomenclatures/classification systems were extended by using the same approach applied to delineate HGs to each pre-existing group in the target system. Newly formed clusters reaching the thresholds for the minimum number of defining genomic variants and minimum total size were reported.

A haplogroup/lineage was considered to originate from a specific country when 50% or more of the genomic sequences included in the haplogroup/lineage were associated with that country, and/or when the same condition applied to the first 50 isolated genomic sequences of the haplogroup. Only sequences for which collection dates were available were included in these analyses.

A Lineage and an HG we considered to be equivalent if they reciprocally shared more than 50% of the sequences assigned to each.

A genomic variant was considered characteristic/defining of a lineage or designation if it was observed in at least 50% of the genomes assigned to that lineage/designation.

### SARS-CoV-2 phylogeny and detection of sites under selection

SARS-CoV-2 phylogeny in Newick format was obtained from the Audacity^48^ tool in GISAID^2^.

Alignments of SARS-CoV-2 protein-coding genes were performed by the means of the Muscle^49^ software. Identification of sites under selection was performed by applying the MEME and FEL methods, as implemented in the Hyphy package^22^, to the phylogeny and the concatenated alignment of protein-coding sequences. A *P* value of 0.05 was considered for the significance threshold. Only sites predicted to be under positive selection according to both methods were considered. For sites predicted to be under negative selection only FEL was used, since MEME can not identify purifying selection^50^.

Phylogenetic analyses of HGs defined by HaploCoV within the AY.4, B.1.1.7, BA.1 and BA.2 Pango lineages were performed by means of the augur pipeline, as implemented by Nexstrain^51^. Genomic positions associated with large insertions or deletions >3 nt in size were masked by the mask-alignment.py as available from https://github.com/nextstrain/augur. For every Pango lineage only the 50 top HGs comprising the highest number of genomic sequences and including more than 1500 high quality sequences were considered; 100 bootstrap replicates were performed by random resampling of 10 complete, high quality genomic sequences. Consensus trees were obtained by means of the Majority consensus algorithm implemented by Dendroscope^52^ v3.5. Graphical representation of the phylogenetic trees were made with Evolview^53^ v3.

### Survival analysis and selection of the highest-scoring prioritization system

The identification of the optimal threshold for the prioritization of SARS-CoV-2 variants was executed by a simple grid search. For every scoring system, scores were computed and the score distribution was obtained. Threshold values were derived by considering the top 200 quantiles of each distribution with a shift of 0.025 (from 99.975 to 85). For every combination the total number designations above of the threshold was computed. The combination of scoring system and threshold value that maximized the total number of VOC/VOI/VUM lineages above the threshold and that, at the same time, minimized the total number non VOC/VOI/VUM lineages above the threshold was selected.

### Statistics and reproducibility

All statistical analyses and tests were performed by means of the stats^54^ R package as provided by the R programming language: Fisher’s exact tests were performed by the fisher.test() function and Wilcoxon rank and sum tests by means of the wilcox.test() function. Dimensionality reduction analyses were performed by the prcomp() function in the R stats package^54^.

### Reporting summary

Further information on research design is available in the Nature Portfolio Reporting Summary linked to this article.

## Supplementary information

Supplementary Information

Description of Additional Supplementary Data

Supplementary Data 1

Supplementary Data 2

Supplementary Data 3

Supplementary Data 4

Supplementary Data 5

Supplementary Data 6

Supplementary Data 7

Supplementary Data 8

Supplementary Data 9

Supplementary Data 10

Supplementary Data 11

Supplementary Data 12

Supplementary Data 13

Supplementary Data 14

Supplementary Data 15

Supplementary Data 16

Supplementary Data 17

Supplementary Data 18

Supplementary Data 19

Supplementary Data 20

Supplementary Data 21

Reporting Summary

## Data Availability

All the data used in this study are publicly available, under restricted access, from the GISAID EpiCoV^2^ database at https://gisaid.org/. Source data underlying Figs. 3, 5 and 6 are presented in Supplementary Data 19–21, respectively.

## Code availability

The software suite for analysis and classification of SARS-CoV-2 genomes described in study is available at: https://github.com/matteo14c/HaploCoV and Zenodo^55^.
