# Supplementary material for: HaploCoV: unsupervised classification and rapid detection of novel emerging variants of SARS-CoV-2
Source: Commun Biol. 2023 Apr 22;6:443. doi: 10.1038/s42003-023-04784-4 (PMC10122080; doi:10.1038/s42003-023-04784-4)
Supplement: Supplementary file 1 — Supplementary Information [file 42003_2023_4784_MOESM1_ESM.pdf]

Supplementary information

Supplementary Data 1: VOC/VOI/VUM as designated by WHO. Ancestor Lineage (Pango): name of the parental lineage according to Pango nomenclature. WHO Label: name according to the simplified WHO nomenclature. Amino acid changes in Spike: list of characteristic non-synonymous substitutions in the Spike glycoprotein. VOC/VOI/VUM: status, VOC (Variant of Concern), VOI (Variant of Interest) or VUM (Variant under Monitoring), with an * used to indicate de-escalated variants. Time first detected: time of isolation of the first genome associated with the lineage. Country of first detection: country where the lineage was originally isolated. Data as of June 10th 2022.

Supplementary Data 2: List of the 238,118 genomic variants identified in SARS-CoV-2 genomic sequences together with their functional annotation. POS: nucleotide position on the reference genome. REF: reference sequence. ALT: alternative sequence. Annot: functional annotation according to CorGAT. AF: Allele Frequency calculated on aggregated data, on overlapping intervals of 10 days starting from December 26th 2019. Uniprot: protein domain according to uniprot annotation of SARS-CoV-2 proteins. HF_cumulative: 1= cumulative AF >1%, 0= cumulative AF<1%. HF_area and HF_country: equivalent to HF_global but with AF data computed at macroarea and at country level.

Supplementary Data 3: Correspondence of countries with geographic macro-areas. Countries are reported in the first column. Geographic macro-areas short and full names are indicated in the second and third columns.

Supplementary Data 4: a) List of high frequency genomic variants associated with distinct geographic macro-areas. Genomic variants are reported in the first column. Geographic areas are provided in the form of a comma separated list. b) List of high frequency genomic variants associated with distinct countries. Genomic variants are reported in the first column. Countries provided in the form of a comma separated list.

Supplementary Data 5: Percentage of high quality genomes sequenced by different countries. Only countries with >5000 genomes were considered. Column1: % based on original criteria defined in Chiara et al 2021. Column2: % based on revised criteria used in the current study. Column 3: difference between 1 and 2.

Supplementary Data 6. Complete list of genomes considered in our analyses, including a selection of metadata derived from the GISAID database and assignment of HGs (last column).

Supplementary Data 7: List of Pango+ lineages formed in the Pango nomenclature by HaploCoV. Pango+ lineages are indicated by the addition of the suffix ".N" to the name of the parental Pango lineage. Parental lineage: Pango designation of the parental lineage. Defining parental: defining genomic variants of the parental lineage. Additional: additional genomic variants of the Pango+ lineage.

Supplementary Data 8: List of defining genomic variants associated with Pango+ lineages. Genomic variants are reported in the first column. The second and third columns report the total number of Pango+ lineages associated with a specific genomic variant and a complete list of the Pango+ lineages.

Supplementary Data 9: Number of countries and macro geographic areas associated with Pango+ lineages. Lineages are reported in the first column. Columns 2 and 3 report the total number of geographic areas from where the lineages were isolated and the complete list (comma separated). Columns 4 and 5 include the equivalent information for countries.

Supplementary Data 10: Presumed country of origin for the Pango and Pango+ lineages and HGs defined by HaploCoV. a) Pango/Pango+ lineages. b) HGs. % supporting genomes: proportion of genomes supporting the reported country of origin. Only the first 50 genomics sequences were considered for every HG/Pango(+) lineage. NA=not assigned.

Supplementary Data 11: Additional groups formed within VOCs/VOIs/VUMs. Ancestral Pango Lineage: ancestral lineage according to the Pango nomenclature. SubLin Pango: total number of derivative lineages in Pango. Additional Pango+: total number of additional designations formed by HaploCoV. Tot genomes in Pango +: total number of genomes assigned to the additional designations formed by our system. Tot genomes in SubLin Pango: total number of genomic sequences assigned to VOC/VOI/VUM sub-lineages in the Pango nomenclature

Supplementary Data 12: List of features used in the classification of SARS-CoV-2 lineages/HGs. id: Acronyms of the considered feature. A brief description is reported in the second column; P-values for the significance of the differences between distributions in VOC/VOI/VUM and other variants are reported in the third column.

Supplementary Data 13: Sites under selection, determined by Hyphy (Kosakovsky-Pond et al23). Residue: amino acid residue. Meme: True/False for the criterion under selection according to Meme. Fel: True/False for the criterion under selection according to Fel. Type: type of selection positive/negative.

Supplementary Data 14: list of genomic variants over-represented in VOC/VOI/VUM Pango lineages. A FDR of 0.05 or below was used as the threshold for significant over-representation. Genomic variants are indicated in the first column. #VOC/VOI/VUM over a total of 28: total number of occurrences in VOC/VOI/VUM. #Others over a total of 1333: total number of occurrences for non VOC/VOI/VUM variants. The p-value and FDR columns report p-valued and adjusted p-values for multiple testing for the over-representation in VOC/VOI/VUM, respectively. The column Annotation reports the functional annotation according to CorGAT.

Supplementary Data 15: Evaluation of SARS-CoV-2 variant prioritization scoring systems. Total # of features included: number of features included in the system. Min Score: minimum score. Max Score: maximum score. Opt Threshold: optimal threshold for the identification of epidemiologically relevant variants as defined by survival analysis. % VOC-VOI-VUM Lin above Threshold (True positives): % of VOC/VOI/VUM associated lineages with a score above the threshold. % Lin of "Others" above Threshold (False positives): % of non VOC/VOI/VUM associated lineages with a score above the threshold. % VOC-VOI-VUM above Threshold (True positives): % of VOC/VOI/VUM variants with a score above the threshold. % other variants above threshold (False positives): % of non VOC/VOI/VUM variantswith a score above the threshold. FDR: false discovery rate corrected p-values for the significance of the difference in score distributions between VOC/VOI/VUM associated lineages and lineages associated with other variants. The optimal scoring system is highlighted in yellow.

Supplementary Data 16: Non VOC/VOI/VUM lineages prioritized by HaploCoV. Lineage (Pango): according to the Pango nomenclature. High prevalence: list of countries/areas (if any) where the lineage reached a prevalence above 1%. Monitored by local health authorities/immune escape: references on lineage/variant previously monitored by local health authorities and/or linked with immune escape . Before Alpha/Beta: 1= the lineage passed the threshold for prioritization only before the emergence of the Alpha or Beta; 0= the lineage was prioritized by the system after the emergence of Alpha/Beta. Zoonotic: evidence for zoonotic transmission.

Supplementary Data 17: Pango+ Lineages showing an increased prioritization score. VOC: VOC to which a Pango Lineage is associated. Pango+ Lineage: name of the Pango+ lineage. Parental: parental lineage in the Pango nomenclature. #defining genomic variants Pango+: number of defining genomic variants for the Pango+ lineage. #defining genomic variants parental: number of defining genomic variants for the parental Pango lineage. Additional Spike NS: list of non-synonymous substitutions specific to the Pango+ lineage in the Spike glycoprotein. Additional non Spike NS: list of non-synonymous substitutions specific to the Pango+ lineage, in protein coding genes other than Spike. First isolate: date and place of isolation of the first isolate. #isolates: total number of isolates associated with the Pango+ lineage. Country max (Pango+): country with the highest number of genomic sequences assigned to the Pango+ lineage. The Pango+ lineages described in Figure 6 are highlighted in red.

Supplementary Data 18: Highly variable Pango+ Lineages showing an increased prioritization score**.** Highly variable Pango+ lineages were defined as having ≥ 6 additional genomic variants with respect to their parental strain and supported by ≥ 5 isolates. Pango+ Lineage: name of the Pango+ lineage. Parental: parental lineage in the Pango nomenclature. #defining genomic variants Pango+: number of defining genomic variants for the Pango+ lineage. #defining genomic variants parental: number of defining genomic variants for the parental Pango lineage. Additional Spike NS: list of non-synonymous substitutions specific to the Pango+ lineage, in the Spike glycoprotein. Additional non Spike NS: list of non-synonymous substitutions specific to the Pango+ lineage, in protein coding genes other than Spike. First isolate: date and place of isolation of the first isolate. Num isolates: total number of isolates associated with the Pango+ lineage. Country max (Pango+): country with the highest number of genomic sequences assigned to the Pango+ lineage.

Supplementary Data 19: Source data for Figure 3.

Supplementary Data 20: Source data for Figure 5.

Supplementary Data 21: The source data for Figure 6.

Supplementary Figure 1

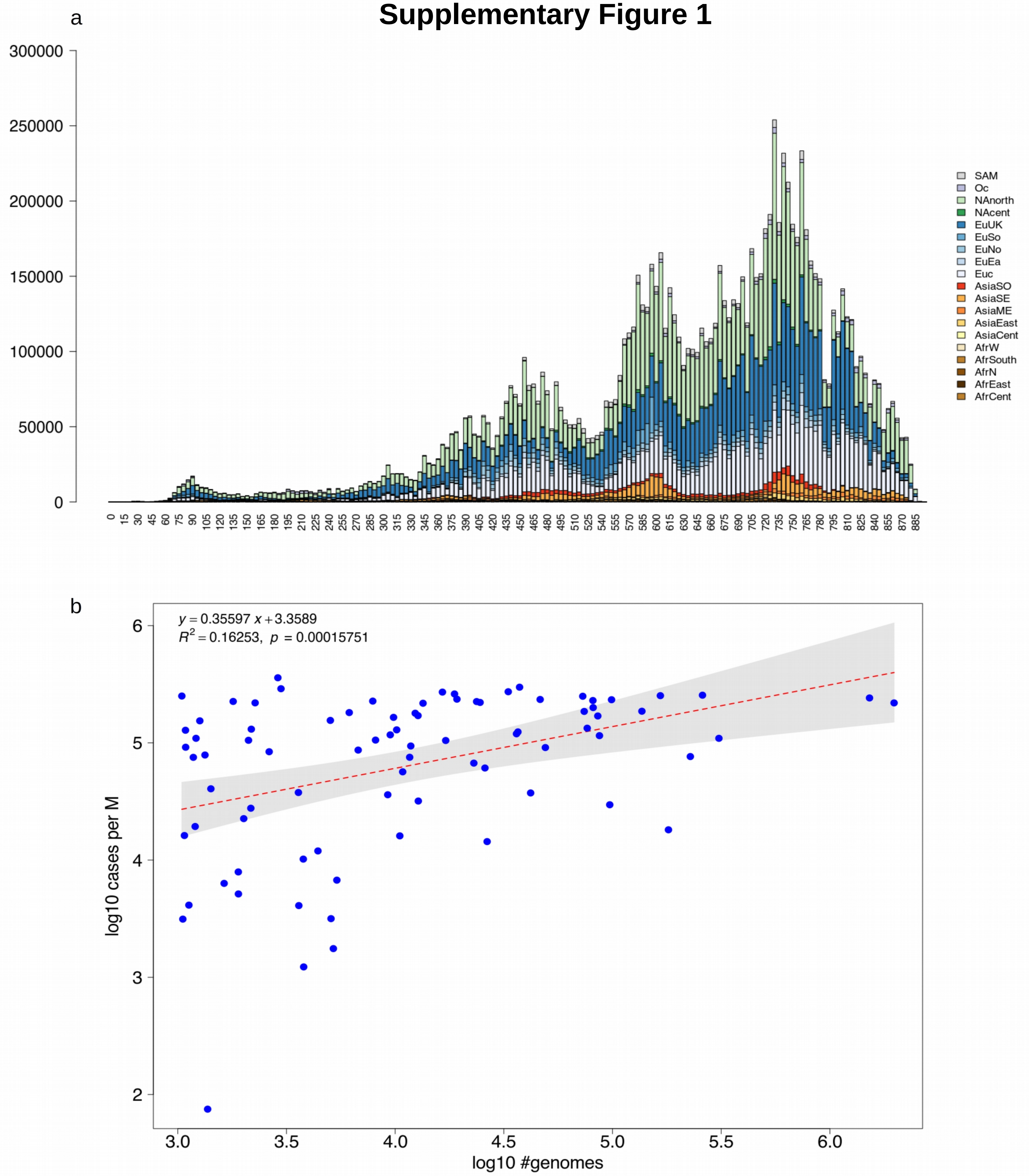

Supplementary Figure 1: Total number of genomic sequences from different geographic areas and correlation with the incidence of COVID-19. a) total number of genomic sequences deposited at intervals of 10 days from different geographic areas. Time T0 sets at Time 0= December 26th 2019, i.e. the day of reported isolation of the first SARS-CoV-2 genomic sequence. The following acronym are used for different geographic areas, as also defined in Supplementary Table S2: AfrCent: Central Africa; AfrEast: Eastern Africa; AfrN: Northern Africa; AfrSouth: Southern Africa; AfrW: Western Africa; AsiaCent: Central Asia; AsiaEast: Eastern Asia; AsiaME: Middle East; AsiaSE: South Eastern Asia; AsiaSO: Southern Asia; Euc: Central Europe; EuEa: Eastern Europe; EuNo: Northern Europe; EuSo: Southern Europe; EuUK: United Kingdom; NAcent: central America; NAnorth: North America; Oc: Oceania; SAM: South America. b) correlation between incidence of COVID-19 (log10 cases per Million, Y axis) and total number of SARS-CoV-2 genomic sequences deposited in public databases (log 10 genomes, x axis) in 100 countries for which at least 1000 genomes of SARS-CoV-2 have been deposited. Regression equation and R2 are shown. The gray area represents a 0.95 level of confidence interval.

Supplementary Figure 2

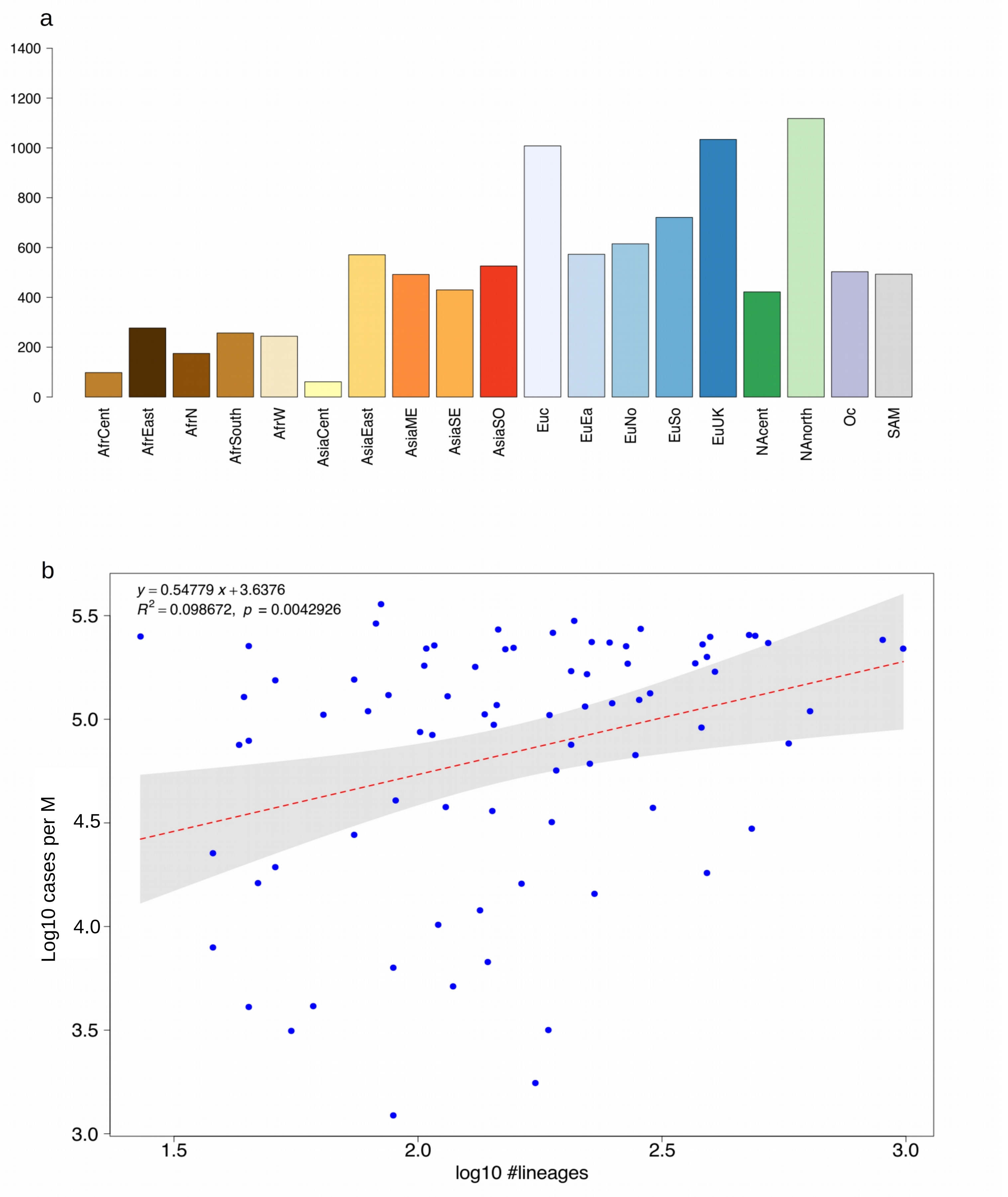

Supplementary Figure 2: a) Barplot of total number of Pango lineages by geographic area. The barplot reports the total number of Pango lineages that reached a frequency of at least 1% in different geographic areas. Acronyms and color code according to Supplementary Figure. b) correlation between incidence of COVID-19 (log10 cases per Million, Y axis) and total number of Pango lineages associated with sequences deposited in public databases (log 10 lineages, x axis). Regression equation and R2 are shown. The gray area represents a 0.95 level of confidence interval.

Supplementary Figure 3

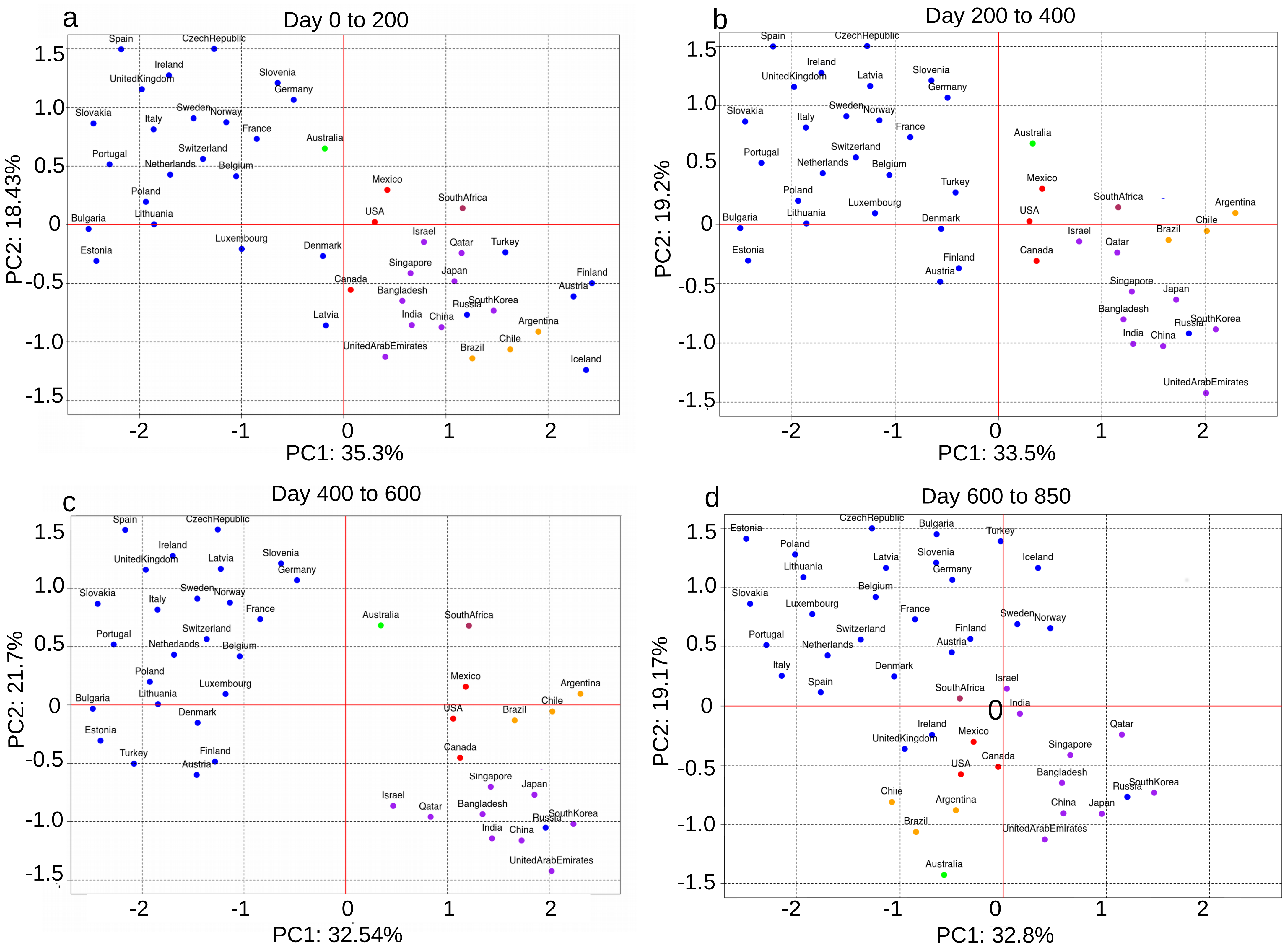

Supplementary Figure 3: Principal component analysis of allele frequencies in different countries for which ≥ 1000 genomic sequences were available. Four distinct time intervals are considered, with time T0 set at December 26th 2019 i.e., the day of reported isolation of the first SARS-CoV-2 genomic sequence. a) from day 0 to 199. b) from day 200 to 399. c) from day 400 to 599, d) from day 600 to 850. Countries of different geographic areas are reported in the following colors: Europe: blue; North America: red; South America: orange; Asia: purple; Australia: green.

Supplementary Figure 4

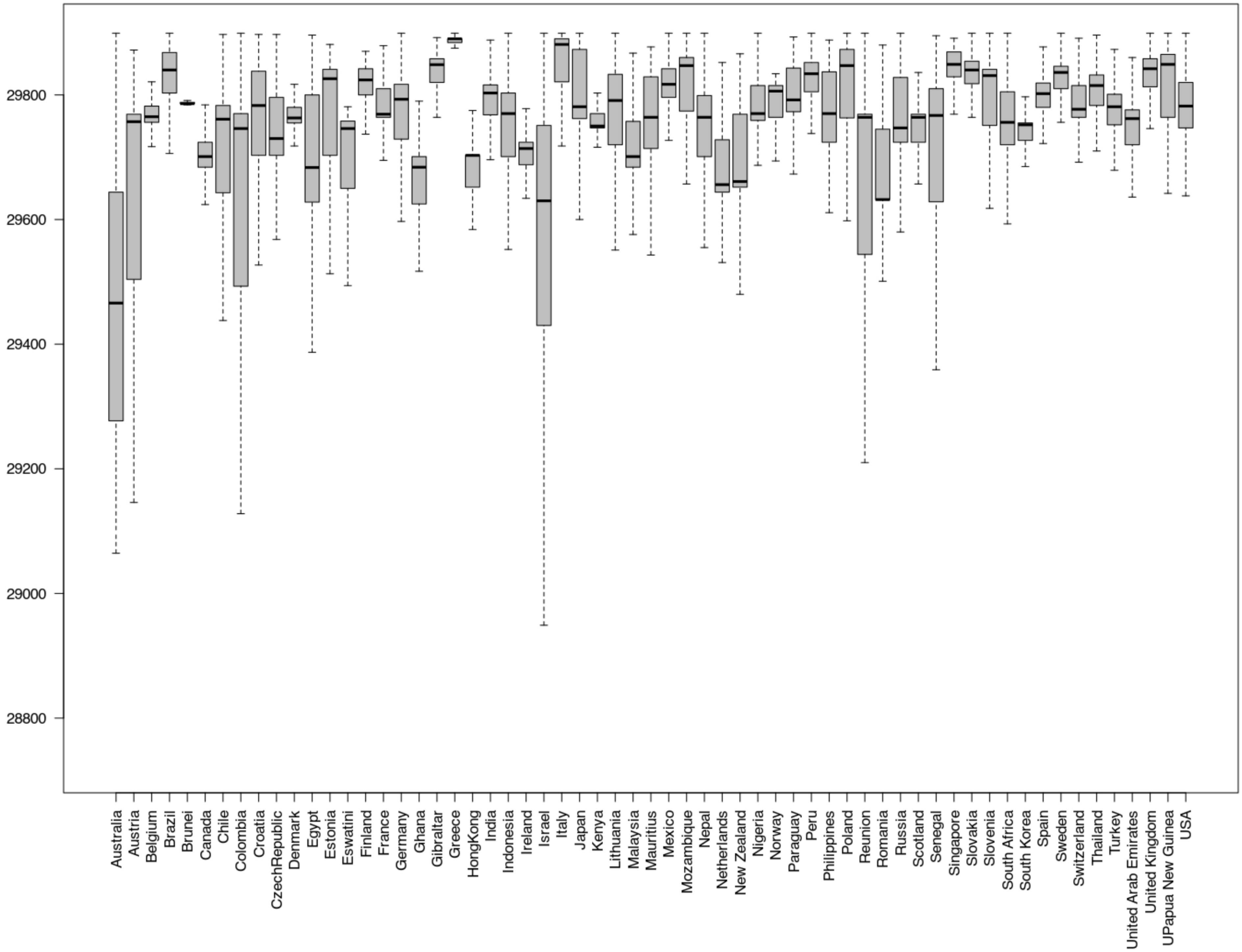

Supplementary Figure 4: Size of genome assembly by country of isolation. Boxplots represent distribution of genome assembly size for 38 distinct countries for which more than 10,000 genome assemblies of SARS-CoV-2 are available in the GISAID database as of June 10th 2022. Countries are indicated on the x axis. Size of assembled genomes is reported on the y axis. In the boxplots, horizontal black lines denote median values; boxes extend from the 25th to the 75th percentile; vertical extending lines denote adjacent values (i.e., the most extreme values within 1.5 interquartile range of the 25th and 75th percentile of each group).

Supplementary Figure 5

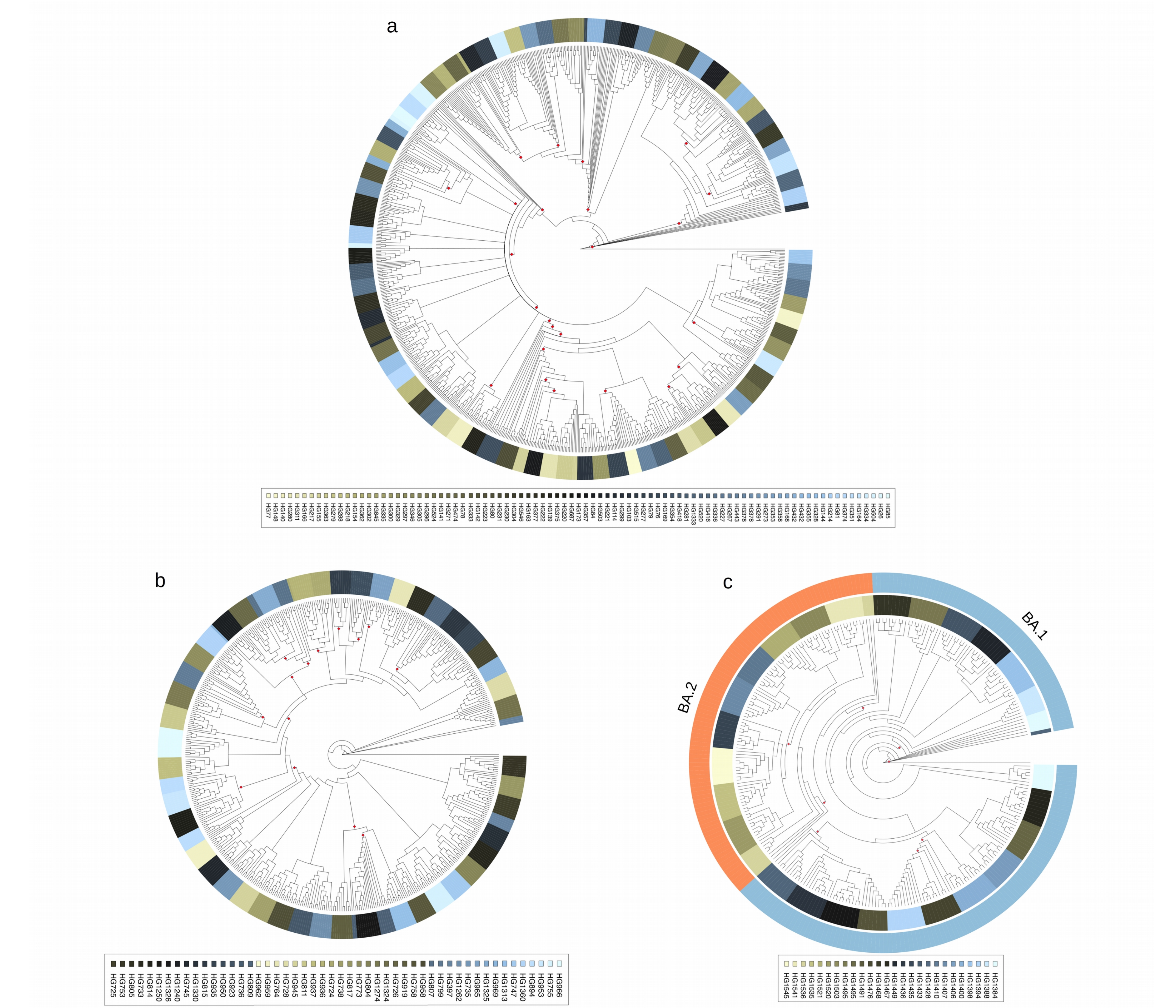

Supplementary Figure 5: Phylogeny of HGs formed by HaploCoV in the most widespread Pango lineages associated with the Alpha, Delta and Omicron VOC. A total of 86, 51 and 27 distinct HGs were considered for Alpha (B.1.1.7 lineage), Delta (AY.4 lineage) and Omicron (BA.2 and BA.1 lineage). Criteria for the inclusion/exclusion of sequences as indicated in Materials and Methods. For every HG 10 representative sequences are reported in the tree, a total of 100 bootstrap replicates were made. Bootstrap values lower than 50 are indicated by a red diamond. a) Alpha, B.1.1.7. b) Delta, AY.4. c) Omicron BA.1 and BA.2.
