# Supplementary material for: HaploCoV: unsupervised classification and rapid detection of novel emerging variants of SARS-CoV-2
Source: Commun Biol. 2023 Apr 22;6:443. doi: 10.1038/s42003-023-04784-4 (PMC10122080; doi:10.1038/s42003-023-04784-4)
Supplement: Supplementary file 2 — Description of Additional Supplementary Data [file 42003_2023_4784_MOESM2_ESM.docx]

**Description of Additional Supplementary Files**

**File name:** Supplementary Data 1

**Description:** VOC/VOI/VUM as designated by WHO.

**File name:** Supplementary Data 2

**Description:** List of the 238,118 genomic variants identified in SARS-CoV-2 genomic sequences together with their functional annotation.

**File name:** Supplementary Data 3

**Description:** Correspondence of countries with geographic macro-areas.

**File name:** Supplementary Data 4

**Description:** List of high frequency genomic variants

**File name:** Supplementary Data 5

**Description:** Percentage of high quality genomes sequenced by different countries

**File name:** Supplementary Data 6

**Description:** Complete list of genomes considered in our analyses

**File name:** Supplementary Data 7

**Description:** List of Pango+ lineages formed in the Pango nomenclature by HaploCoV.

**File name:** Supplementary Data 8

**Description:** List of defining genomic variants associated with Pango+ lineages.

**File name:** Supplementary Data 9

**Description:** Number of countries and macro geographic areas associated with Pango+ lineages

**File name:** Supplementary Data 10

**Description:** Presumed country of origin for the Pango and Pango+ lineages and HGs defined by HaploCoV.

**File name:** Supplementary Data 11

**Description:** Additional groups formed within VOCs/VOIs/VUMs by HaploCoV

**File name:** Supplementary Data 12

**Description:** List of features used in the classification of SARS-CoV-2 lineages/HGs..

**File name:** Supplementary Data 13

**Description:** Sites under selection, determined by Hyphy

**File name:** Supplementary Data 14

**Description:** list of genomic variants over-represented in VOC/VOI/VUM Pango lineages.

**File name:** Supplementary Data 15

**Description:** Evaluation of SARS-CoV-2 variant prioritization scoring systems.

**File name:** Supplementary Data 16

**Description:** Non VOC/VOI/VUM lineages prioritized by HaploCoV.

**File name:** Supplementary Data 17

**Description:** Pango+ Lineages showing an increased prioritization score..

**File name:** Supplementary Data 18

**Description:** Highly variable Pango+ Lineages showing an increased prioritization score.

**File name:** Supplementary Data 19

**Description:** The source data behind Figure 3

**File name:** Supplementary Data 20

**Description:** The source data behind Figure 5

**File name:** Supplementary Data 21

**Description:** The source data behind Figure 6
